# DCAF7 Acts as A Scaffold to Recruit USP10 for G3BP1 Deubiquitylation and Facilitates Chemoresistance and Metastasis in Nasopharyngeal Carcinoma

**DOI:** 10.1002/advs.202403262

**Published:** 2024-07-08

**Authors:** Qing‐Jie Li, Xue‐Liang Fang, Ying‐Qin Li, Jia‐Yi Lin, Cheng‐Long Huang, Shi‐Wei He, Sheng‐Yan Huang, Jun‐Yan Li, Sha Gong, Na Liu, Jun Ma, Yin Zhao, Ling‐Long Tang

**Affiliations:** ^1^ Sun Yat‐sen University Cancer Center State Key Laboratory of Oncology in South China Collaborative Innovation Center of Cancer Medicine Guangdong Key Laboratory of Nasopharyngeal Carcinoma Diagnosis and Therapy 651 Dongfeng Road East Guangzhou Guangdong 510060 China

**Keywords:** chemoresistance, chemotherapy, DCAF7, deubiquitylation, nasopharyngeal carcinoma

## Abstract

Despite docetaxel combined with cisplatin and 5‐fluorouracil (TPF) being the established treatment for advanced nasopharyngeal carcinoma (NPC), there are patients who do not respond positively to this form of therapy. However, the mechanisms underlying this lack of benefit remain unclear. DCAF7 is identified as a chemoresistance gene attenuating the response to TPF therapy in NPC patients. DCAF7 promotes the cisplatin resistance and metastasis of NPC cells in vitro and in vivo. Mechanistically, DCAF7 serves as a scaffold protein that facilitates the interaction between USP10 and G3BP1, leading to the elimination of K48‐linked ubiquitin moieties from Lys76 of G3BP1. This process helps prevent the degradation of G3BP1 via the ubiquitin‒proteasome pathway and promotes the formation of stress granule (SG)‐like structures. Moreover, knockdown of G3BP1 successfully reversed the formation of SG‐like structures and the oncogenic effects of DCAF7. Significantly, NPC patients with increased levels of DCAF7 showed a high risk of metastasis, and elevated DCAF7 levels are linked to an unfavorable prognosis. The study reveals DCAF7 as a crucial gene for cisplatin resistance and offers further understanding of how chemoresistance develops in NPC. The DCAF7‐USP10‐G3BP1 axis contains potential targets and biomarkers for NPC treatment.

## Graphical abstract

DCAF7 is identified as a key chemoresistance gene enhancing cisplatin resistance and metastasis in NPC, serving as a scaffold that recruits USP10 to eliminate K48‐linked ubiquitin moieties from Lys76 of G3BP1, thus promoting the formation of stress granule‐like structures. High levels of DCAF7 correlate with metastasis risk and poor prognosis, providing potential targets for overcoming NPC chemoresistance.

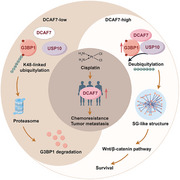

## 1 Introduction

Nasopharyngeal carcinoma (NPC), a type of cancer that affects the head and neck region, is commonly found in Southeast Asia, particularly in South China.^[^ ^1^ ^]^ Currently, docetaxel plus cisplatin and 5‐fluorouracil (TPF) induction chemotherapy is the standard regimen for patients with locoregionally advanced NPC (LA‐NPC).^[^ ^2^ ^]^ However, ≈10% of NPC patients have unsatisfactory outcomes after receiving this treatment, owing primarily to the emergence of chemoresistance.^[^ ^3^ ^]^ Therefore, elucidating the molecular mechanisms underlying chemoresistance is crucial and may identify potential targets for sensitizing patients with LA‐NPC to chemotherapy.

Scaffold proteins compose a class of proteins (more than 300 proteins) that act as molecular hubs for the docking of other proteins to organize functional units for signalling cascades.^[^ ^4^ ^]^ Scaffold proteins interact with multiple binding partners and play a role in various biological processes, such as cell cycle, cell growth, immune response, and restructuring of the cytoskeleton.^[^ ^5^ ^]^ Dysregulation of scaffold proteins can lead to a wide range of diseases (e.g., cancer, diabetes, neutropenia and Alzheimer's disease).^[^ ^6^ ^]^ Notably, the regulation of ubiquitination is essential for the control of scaffold protein‐mediated signalling cascades.^[^ ^7^ ^]^ For example, upon ubiquitination, NEMO undergoes conformational changes and liquid–liquid phase separation (LLPS), which increases its binding affinity for other substrates.^[^ ^8^ ^]^ P62 is polyubiquitinated to form LLPS droplets, enabling its involvement in the regulation of cell growth and inflammation.^[^ ^9^ ^]^ TRAF2 recruits cIAP1/2 to ubiquitinate IKKε, causing the activation of NF‐kB signalling.^[^ ^10^ ^]^ ZMIZ2 recruits USP7 to deubiquitinate and stabilize *β*‐catenin.^[^ ^11^ ^]^ DCAF7 is a scaffold protein that belongs to the DDB1 and CUL4‐associated factor (DCAF) family, whose members often serve as E3 ubiquitin ligase substrate receptors and regulate various signalling cascades and events, including the YAP‐Hippo pathway, histone methylation, and HIPK2‐MAPK signalling.^[^ ^12^ ^]^ However, whether DCAF7 plays a role in NPC tumorigenesis or progression has not been determined.

Stress granules (SGs) are formed when large amounts of mRNAs, RNA‐binding proteins, and translation initiation factors undergo cytoplasmic condensation in response to stressful conditions, such as heat shock, osmotic pressure, and drug exposure.^[^ ^13^ ^]^ Ras GTPase‐activating protein‐binding protein 1 (G3BP1) and its interactions with USP10 and Caprin1 are important for SG formation and regulation.^[^ ^14^ ^]^ G3BP1 modulates a range of cellular processes (e.g., mRNA stability, rasGAP signalling, ubiquitination, and mRNA metabolism) in response to intracellular and extracellular stimuli through its interactions with RNA and proteins, and dysregulation of these interaction leads to neurological disorders, cancer progression, and bacterial and viral infections.^[^ ^15^ ^]^ USP10 interacts with G3BP1 and prevents the recruitment of other RNAs and proteins for SG formation.^[^ ^16^ ^]^ However, whether G3BP1‐mediated SGs contain other binding partners and how these potential binding partners interact with USP10 and G3BP1 remain largely unknown.

Herein, we found that DCAF7 functions as a framework for bringing USP10 to G3BP1 and further deubiquitinates and stabilizes this protein, thus promoting the G3BP1‐mediated formation of SG‐like structures. Specifically, DCAF7 was found to be highly expressed in TPF‐resistant NPC patients and to promote the cisplatin resistance and metastasis of NPC cells. Further mass spectrometry (MS) analysis identified G3BP1 and USP10 as binding partners of DCAF7. Mechanistically, DCAF7 promoted the binding of USP10 to G3BP1, leading to the elimination of K48‐linked ubiquitin moieties from Lys76 of G3BP1, thus preventing the degradation of G3BP1 via the ubiquitin‒proteasome pathway and facilitating SG‐like structures to form. In addition, knockdown of G3BP1 reversed the formation of SG‐like structures as well as the oncogenic effects of DCAF7. Significantly, NPC patients with increased level of DCAF7 showed a high risk of metastasis and a poor prognosis. Our study identifies a novel binding partner for G3BP1 and broadens the understanding of G3BP1‐mediated formation of SG‐like structures. More importantly, we identify DCAF7 crucial gene for cisplatin resistance and gain insights into the mechanisms that contribute to TPF resistance in NPC patients, thus identifying potential therapeutic targets for NPC.

## 2 Results

### 2.1 DCAF7 Expression is Associated with Chemoresistance and Indicates a Poor Prognosis

**Figure 1:**
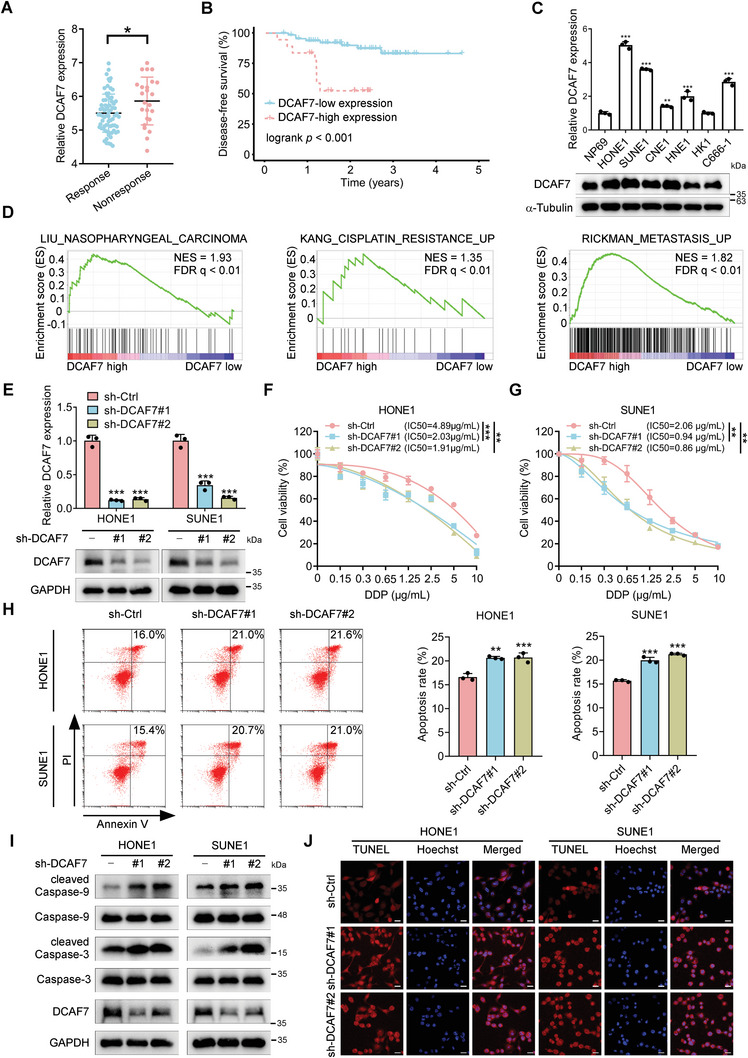
DCAF7 is associated with chemoresistance and indicates a poor prognosis. A) mRNA expression levels of *DCAF7* in NPC patients who received TPF chemotherapy, based on the GSE132112 dataset. Student's *t*‐test, ^*^ *p* < 0.05. B) Kaplan–Meier survival analysis of patients with NPC in the GSE102349 dataset (n = 88) stratified by DCAF7 expression (high vs low). C) RT‒qPCR and western blot analysis results showing the mRNA and protein expression levels, respectively, of DCAF7 in NPC and NP69 cells. Mean (n = 3) ± s.d. One‐way ANOVA, ^**^ *p* < 0.01, ^***^ *p* < 0.001. D) GSEA of the GSE102349 dataset revealed positive enrichment of genes associated with NPC, cisplatin resistance and metastasis signatures in response to high DCAF7 expression. E) The DCAF7 knockdown efficiency was assessed using RT‒qPCR and western blotting. Mean (n = 3) ± s.d. One‐way ANOVA, ^***^ *p* < 0.001. F,G) A CCK‐8 assay was used to evaluate cisplatin resistance in transfected NPC cells following treatment with the indicated concentrations of cisplatin for 48 h. Mean (n = 4) ± s.d. Two‐way ANOVA, ^**^ *p* < 0.01, ^***^ *p* < 0.001. H) NPC cells were exposed to cisplatin (2.5 µg mL^−1^) for 24 h, and cisplatin‐induced apoptosis was measured via Annexin‐V/PI staining and flow cytometry. Mean (n = 3) ± s.d. One‐way ANOVA, ^**^ *p* < 0.01, ^***^ *p* < 0.001. I) NPC cells were treated with cisplatin (10 µg mL^−1^) for 24 h. The levels of apoptosis‐related proteins, including Caspase3/9 and cleaved Caspase3/9, were measured via western blotting. J) NPC cells were treated with cisplatin (10 µg mL^−1^) for 24 h, and cisplatin‐induced apoptosis was detected using a TUNEL assay. Scale bars = 20 µm. The unprocessed images of the blots are shown in Figure S10 (Supporting Information).

Our previous study demonstrated that TPF chemotherapy can significantly improve the survival of NPC patients.^[^ ^2b^ ^]^ However, some patients experience recurrence or metastasis after TPF chemotherapy. We then found a set of mRNAs that can predict the efficacy of TPF chemotherapy by microarray sequencing.^[^ ^17^ ^]^ To further explore the mechanisms underlying TPF resistance, we divided patients who received TPF chemotherapy into the response and nonresponse groups and found that DCAF7 was significantly upregulated in the nonresponse group (**Figure** **1**A). Following this, NPC patients who exhibited elevated level of DCAF7 had a worse prognosis compared to those with lower level of DCAF7 (Figure 1B). It is worth mentioning that DCAF7 showed increased levels in nearly all solid tumors compared to the normal tissues, with elevated DCAF7 expression correlating with a negative prognosis (Figure S1A–E, Supporting Information), suggesting a significant involvement of DCAF7 in cancer development. A previous study has revealed that DCAF7 leads to resistance to the mTOR inhibitor everolimus in pancreatic neuroendocrine tumors.^[^ ^18^ ^]^ However, there are currently no reports detailing the functional role of DCAF7 in tumor metastasis, and the precise biological function and molecular mechanisms of DCAF7 in the pathogenesis of NPC remain to be elucidated.

To explore the function of DCAF7 in the progression of NPC, we analyzed the levels of its mRNA and protein expression in various NPC cell lines and normal nasopharyngeal cells (NP69). Figure 1C demonstrated that NPC cells exhibited elevated levels of DCAF7 expression compared to NP69 cells, suggesting a potential role for DCAF7 in NPC. To support these findings, we conducted gene set enrichment analysis (GSEA) and found a strong correlation between elevated DCAF7 levels and the presence of gene patterns linked to cisplatin resistance and metastasis (Figure 1D). Collectively, these results suggest that DCAF7 is associated with poor prognosis and imply its potential role in regulating metastasis and cisplatin resistance in NPC.

### 2.2 DCAF7 Facilitates Cisplatin Resistance in NPC Cells In Vitro

Given that DCAF7 is associated with cisplatin resistance, as shown by the GSEA results in Figure 1D, we then generated DCAF7‐overexpressing and DCAF7‐knockdown NPC cells (Figure 1E; Figure S2A, Supporting Information) and treated these cells with cisplatin. Cell Counting Kit‐8 (CCK‐8) assays demonstrated a notable decrease in the IC50 value in the DCAF7‐knockdown group compared to the control group (Figure 1F,G). Additionally, given the high expression of DCAF7 in NPC tissues resistant to the TPF induction chemotherapy regimen, we then examined whether DCAF7 has impacts on docetaxel and 5‐fluorouracil resistance. The results indicated that the knockdown of DCAF7 did not significantly affect the sensitivity of NPC cells to docetaxel and 5‐fluorouracil (Figure S2C–E, Supporting Information). These findings suggest that the influence of DCAF7 on chemotherapy resistance is mainly specific to cisplatin within the TPF regimen.

In line with the function of DCAF7 in cisplatin resistance, flow cytometry revealed that reducing DCAF7 levels led to a higher percentage of cells undergoing apoptosis after exposure to cisplatin (Figure 1H). Considering the cleaved caspase‐3 and ‐9 are well‐known biomarkers for cell death by apoptosis,^[^ ^19^ ^]^ we then checked the expression of cleaved caspase‐3 and ‐9 upon cisplatin treatment. The results revealed that there was a notable rise in the levels of cleaved caspase 3 and caspase 9 expression in DCAF7‐knockdown NPC cells when exposed to cisplatin in comparison to the original cells (Figure 1I). Terminal deoxynucleotidyl transferase nick‐end labelling (TUNEL) assays were performed to examine DNA damage in NPC cells treated with cisplatin, revealing a significant rise in TUNEL‐positive cells after cisplatin treatment due to DCAF7 knockdown (Figure 1J). The findings suggest that reducing DCAF7 levels increases the cisplatin sensitivity of NPC cells. However, upregulation of DCAF7 decreased the cisplatin sensitivity of NPC cells, as evidenced by the higher IC50 value, reduced apoptosis, lower levels of cleaved caspase 3 and caspase 9, and fewer TUNEL‐positive cells following cisplatin treatment (Figure S2F–H, Supporting Information). Taken together, our results demonstrate that DCAF7 facilitates cisplatin resistance in NPC cells in vitro.

### 2.3 DCAF7 Facilitates NPC Cell Migration, Invasion and Epithelial–Mesenchymal Transition (EMT)

In order to investigate the impact of DCAF7 on NPC cell metastasis as suggested by the GSEA findings (Figure 1D), we performed Transwell assays in DCAF7‐knockdown NPC cells. As expected, knockdown of DCAF7 suppressed the migration and invasion of NPC cells (Figure S3A, Supporting Information), while overexpression of DCAF7 had the opposite effects (Figure S3B, Supporting Information). Given the well‐established role of EMT in malignant progression and metastasis,^[^ ^20^ ^]^ we examined whether DCAF7 influences EMT in NPC cells. DCAF7 knockdown led to higher level of the epithelial marker E‐cadherin protein and lower level of the mesenchymal marker Vimentin protein (Figure S3C, Supporting Information). Conversely, overexpression of DCAF7 had the opposite effects (Figure S3C, Supporting Information). The immunofluorescence (IF) assay results further validated the above findings (Figure S3D,E, Supporting Information). Overall, these findings indicate that DCAF7 plays a role in promoting NPC cell migration, invasion, and epithelial‐mesenchymal transition.

### 2.4 Knockdown of DCAF7 Increases the Chemosensitivity and Suppresses the Metastasis of NPC Cells In Vivo

**Figure 2:**
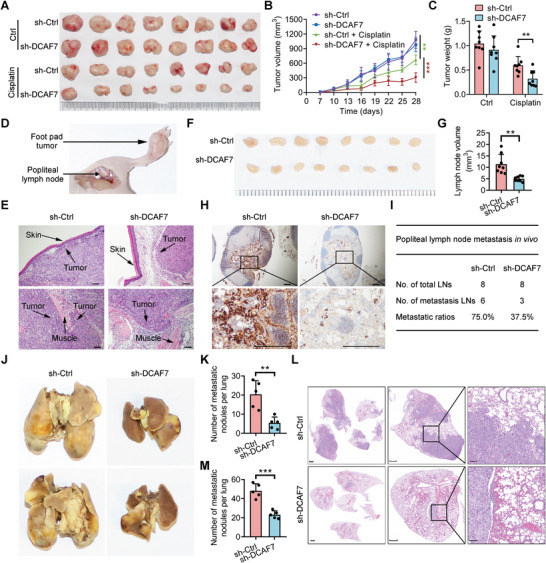
Knockdown of DCAF7 increases the chemosensitivity and suppresses the metastasis of NPC cells in vivo. A) Representative images displaying xenografts in nude mice. B,C) Growth curves and weights of xenograft tumors subjected to the indicated treatments. Mean (n = 8) ± s.d. Two‐way ANOVA in B, Student's *t*‐test in C, ^**^ *p* < 0.01, ^***^ *p* < 0.001. D) Schematic representation of the in vivo lymphatic metastasis model. E) Representative images of H&E‐stained footpad tumors; scale bars = 100 µm. F,G) Representative images and quantitative data for lymph nodes (n = eight mice per group). Mean (n = 8) ± s.d. Student's *t*‐test, ^**^ *p* < 0.01. H,I) IHC images of popliteal lymph nodes stained with an anti‐pancytokeratin antibody, along with the corresponding metastasis rates. Scale bars = 500 µm. J,K) Representative images and quantification of macroscopic lung surface metastatic foci. Mean (n = 8) ± s.d. Student's *t*‐test, ^**^ *p* < 0.01. L,M) Histological images of H&E‐stained lung tissue sections and quantification of microscopic lung metastatic foci. Scale bars = 1000 µm (left), 500 µm (middle), 100 µm (right). Mean (n = 8) ± s.d. Student's *t*‐test, ^***^ *p* < 0.001.

To further evaluate the function of DCAF7 in chemoresistance and metastasis in NPC, we used subcutaneous xenograft, popliteal lymph node and lung metastasis mouse models. In the subcutaneous xenograft mouse model, knockdown of DCAF7 significantly enhanced cisplatin sensitivity, leading to reduced tumor size and weight in cisplatin‐treated mice (**Figure** **2**A–C). Compared to the control group, the tumor growth inhibition (TGI) was observed to be 39.86% (*P* < 0.01) following cisplatin monotherapy (Figure 2B). However, the combination of DCAF7 knockdown with cisplatin markedly enhanced the tumor growth inhibition, achieving a TGI of 74.63% (*P* < 0.001) in NPC in vivo at the termination of the experiment (Figure 2B). In the popliteal lymph node metastasis mouse model, footpad tumors in the DCAF7‐knockdown group showed a less aggressive phenotype, with a decreased invasion of NPC cells into the skin and muscle (Figure 2D,E). Moreover, the DCAF7‐knockdown group exhibited reduced size of lymph nodes compared to the control group (Figure 2F,G). Further immunohistochemical analysis showed a notable reduction in the rate of popliteal lymph node metastasis in the DCAF7‐knockdown group (Figure 2H,I). Furthermore, DCAF7 knockdown significantly decreased the quantity of lung nodules in the lung metastasis mouse model (Figure 2J–M). These findings demonstrate that DCAF7 positively regulates NPC progression in vivo.

### 2.5 Knockdown of DCAF7 Facilitates the Degradation of G3BP1 by Increasing Its K48‐Linked Polyubiquitination

**Figure 3:**
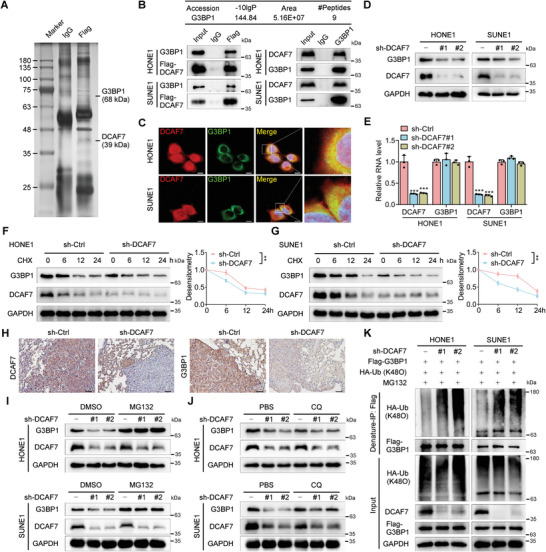
Knockdown of DCAF7 facilitates the degradation of G3BP1 by increasing its K48‐linked polyubiquitination. A) Flag‐DCAF7‐ or vector‐transfected SUNE1 cells were subjected to immunoprecipitation with an anti‐Flag antibody, followed by SDS‒PAGE and silver staining of proteins. The proteins in the bands were analyzed by MS. B) The mass spectrometry results identifying G3BP1 as a potential binding partner of DCAF7 (top). Immunoprecipitation (IP) with an anti‐Flag or anti‐G3BP1 antibody and immunoblot analysis (IB) of G3BP1, Flag or DCAF7 expression in HONE1 and SUNE1 cells transfected with or without Flag‐DCAF7 (bottom). C) Confocal microscopy images showing the colocalization of DCAF7 and G3BP1 in HONE1 and SUNE1 cells. Scale bars: 10, 2 µm (magnified graphs). D,E) Western blotting and RT‒qPCR were used to measure the protein and mRNA levels of G3BP1 in HONE1 and SUNE1 cells following DCAF7 knockdown. Mean (n = 3) ± s.d. One‐way ANOVA, ^***^ *p* < 0.001. F,G) IB of G3BP1, DCAF7 and GAPDH (left) in HONE1 and SUNE1 cells transduced with sh‐DCAF7 or sh‐control following CHX treatment for the indicated times. Plots showing the normalized G3BP1 levels are also presented (right). Mean (n = 3) ± s.d. Two‐way ANOVA, ^**^ *p* < 0.01. H) IHC staining for DCAF7 and G3BP1 in the lung metastatic nodules of the mouse model. Scale bar: 50 µm. I,J) IB of G3BP1, DCAF7 and GAPDH in HONE1 and SUNE1 cells transduced with sh‐control or sh‐DCAF7 following treatment with MG132 (10 µm) or CQ (50 µm). K) Denaturing IP (with an anti‐Flag antibody) and IB of HA, Flag, DCAF7 and GAPDH in HONE1 and SUNE1 cells transfected with the indicated plasmids following MG132 treatment (10 µm, 6 h). The unprocessed images of the blots are shown in Figure S10 (Supporting Information).

To explore the specific mechanisms of DCAF7 in NPC progression, we conducted coimmunoprecipitation (co‐IP) followed by MS (IP–MS) to identify the potential targets of DCAF7 (**Figure** **3**A). Through this IP–MS analysis, 115 proteins were identified (Table S1, Supporting Information), among which 6 protein clusters, including 80 core proteins, were further identified via the STRING database and Cytoscape MCODE plug‐in. The cluster containing G3BP1 was the highest ranked cluster, and the G3BP1 protein was located at the centre of this cluster (Figure S4, Supporting Information). G3BP1, a SG assembly factor, has been shown to be significant in the SG formation and cancer progression.^[^ ^21^ ^]^ We thus selected G3BP1 for further validation. Co‐IP assays further confirmed that both exogenous and endogenous DCAF7 interacted with G3BP1 in NPC cells (Figure 3B). Additionally, this interaction was verified by the colocalization of DCAF7 and G3BP1 in the cytoplasm shown by IF staining (Figure 3C). Since DCAF7 has been reported to be a scaffold protein that recruits the E3 ligase mediating the degradation of its target protein,^[^ ^18^ ^]^ we examined whether DCAF7 affects the expression of G3BP1. Knockdown of DCAF7 dramatically reduced G3BP1 protein expression, whereas overexpression of DCAF7 elevated G3BP1 protein expression (Figure 3D; Figure S5A, Supporting Information). In addition, the mRNA expression level of *G3BP1* did not significantly change with either knockdown or overexpression of DCAF7 (Figure 3E; Figure S5B, Supporting Information). Correspondingly, after treatment with cycloheximide (CHX), DCAF7 knockdown facilitated but DCAF7 overexpression suppressed the degradation of endogenous G3BP1 (Figure 3F,G; Figure S5C,D, Supporting Information), suggesting that DCAF7 prolongs the half‐life of the G3BP1 protein. Specifically, IHC staining revealed a reduction of G3BP1 expression in the lung metastatic nodules of the DCAF7‐knockdown group compared with those in the control group. These results demonstrate that DCAF7 inhibits the degradation of the G3BP1 protein in NPC cells.

To determine whether DCAF7 is involved in the degradation of G3BP1 via either the ubiquitin‒proteasome pathway or the autophagy–lysosomal pathway, NPC cells were exposed to a proteasome inhibitor (MG132) or a lysosome inhibitor (chloroquine; CQ). MG132, but not CQ, reversed the reduction in G3BP1 protein levels caused by DCAF7 knockdown (Figure 3I,J), confirming that DCAF7‐mediated degradation of G3BP1 occurs through the ubiquitin‒proteasome pathway. We then examined the effect of DCAF7 on the ubiquitination of G3BP1 and found that knockdown of DCAF7 increased the K48‐linked, instead of K63‐linked, polyubiquitination of G3BP1 (Figure 3K; Figure S5E, Supporting Information). In summary, these findings demonstrate that DCAF7 increases the stability of G3BP1 by inhibiting its K48‐linked polyubiquitination.

### 2.6 DCAF7 Recruits USP10 to Deubiquitylate and Stabilize G3BP1

**Figure 4:**
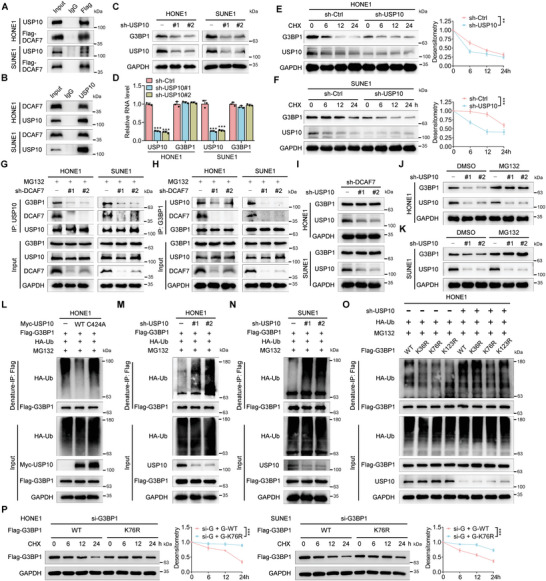
DCAF7 recruits USP10 to deubiquitylate and stabilize G3BP1. A,B) IP (with an anti‐FLAG antibody or IgG) was conducted to validate the interaction between DCAF7 and USP10 in SUNE1 and HONE1 cells transfected with Flag‐DCAF7. C,D) Protein and mRNA levels of G3BP1 in HONE1 and SUNE1 cells with or without USP10 knockdown. Mean (n = 3) ± s.d. One‐way ANOVA, ^***^ *p* < 0.001. E,F) Protein level of G3BP1 in HONE1 and SUNE1 cells with or without USP10 knockdown following CHX treatment (100 µg mL^−1^) for the indicated times. Mean (n = 3) ± s.d. Two‐way ANOVA, ^**^ *p* < 0.01. G,H) IP (with an anti‐USP10 or anti‐G3BP1 antibody) and IB of G3BP1, DCAF7 and USP10 in HONE1 and SUNE1 cells transduced with sh‐control or sh‐DCAF7 following MG132 treatment (10 µm, 6 h). I) IB of G3BP1, USP10 and GAPDH in DCAF7‐knockdown HONE1 and SUNE1 cells transduced with sh‐control or sh‐USP10. J,K) IB of G3BP1, USP10 and GAPDH in HONE1 and SUNE1 cells transduced with sh‐control or sh‐USP10 following MG132 treatment (10 µm, 6 h). L–N) Denaturing IP with an anti‐Flag antibody and IB of HA‐Ub, Flag‐G3BP1, Myc‐USP10 and GAPDH in HONE1 and SUNE1 cells transfected with the indicated plasmids following MG132 treatment (10 µm, 6 h). O) Denaturing IP with an anti‐Flag antibody and IB of HA‐Ub, Flag‐G3BP1, USP10 and GAPDH in HONE1 cells transfected with the indicated plasmids following MG132 treatment (10 µm, 6 h). P) Protein level of Flag‐G3BP1 in HONE1 and SUNE1 cells transfected with indicated siRNA and plasmids following CHX treatment (100 µg mL^−1^) for the indicated times. Mean (n = 3) ± s.d. Two‐way ANOVA, ^**^ *p* < 0.01. The unprocessed images of the blots are shown in Figure S10 (Supporting Information).

The above findings suggest that DCAF7 suppresses the ubiquitination of G3BP1 and thus stabilizes it. We next sought to determine whether DCAF7 can recruit a binding partner, such as a deubiquitinating enzyme, to stabilize G3BP1. Encouragingly, and surprisingly, we found that USP10, a ubiquitin‐specific protease belonging to the deubiquitinating enzyme family that has been reported to interact with G3BP1,^[^ ^16^ ^]^ was among the most common potential interacting proteins of DCAF7 according to previous IP–MS results (Table S1, Supporting Information). Further co‐IP assays confirmed that DCAF7 could interact with both exogenous and endogenous USP10 (**Figure** **4**A,B). Next, we examined whether USP10 affects the stability of G3BP1. Unexpectedly, knockdown of USP10 markedly reduced the protein levels of G3BP1, without affecting its mRNA levels, while overexpression of USP10 led to increased protein levels of G3BP1, with no impact on its mRNA level (Figure 4C,D; Figure S6A,B, Supporting Information). Additionally, the half‐life of the G3BP1 protein was significantly shorter in USP10‐knockdown cells (Figure 4E,F), consistent with the observations in DCAF7‐knockdown cells (Figure 3F,G). Conversely, overexpression of USP10 prolonged the half‐life of the G3BP1 protein (Figure S6C,D, Supporting Information). We then determined whether USP10 is recruited by DCAF7 to stabilize G3BP1 and found that DCAF7 deficiency markedly inhibited the interaction between USP10 and G3BP1 (Figure 4G,H), indicating that DCAF7 is required for this interaction. Moreover, in DCAF7‐knockdown NPC cells, the decrease in G3BP1 expression mediated by USP10 knockdown was completely abolished (Figure 4I), indicating that DCAF7 functions as a scaffold to recruit USP10 for stabilizing G3BP1.

Further investigation confirmed that MG132 treatment restored the expression of G3BP1 in USP10‐knockdown cells (Figure 4J,K). Consistent with these findings, overexpression of USP10 decreased the ubiquitination of G3BP1, while overexpression of a catalytically inactive USP10 mutant (Cys424Ala; C424A)^[^ ^22^ ^]^ had no such effect (Figure 4L), indicating that the deubiquitylase activity of USP10 is essential for G3BP1 deubiquitylation. Furthermore, DCAF7 knockdown resulted in a significant increase in G3BP1 ubiquitination (Figure 4M,N). Since Cindy et al. profiled proteome‐wide ubiquitination sites in USP10‐overexpressing cells and identified three lysine residues as potential ubiquitination sites in G3BP1,^[^ ^23^ ^]^ we constructed three Lys/Arg (K/R) substitution mutants of G3BP1 for denaturing IP assays. Both the K36R and K76R G3BP1 mutants were ubiquitinated less effectively than the wild‐type (WT) G3BP1 in cells without USP10 knockdown, suggesting that G3BP1 can be ubiquitinated at K36 and K76. Correspondingly, the ubiquitination of WT G3BP1 and the K36R mutant was significantly enhanced by USP10 knockdown, while the ubiquitination of the K76R mutant was unaffected by USP10 knockdown (Figure 4O), indicating that USP10 mainly removes the polyubiquitin chain from K76 of G3BP1. To investigate whether the G3BP1 K76R mutant would affect the stability of G3BP1, we conducted western blotting assay in HONE1 and SUNE1 cells that transfected with siRNA targeting G3BP1 (Figure S7A, Supporting Information) and then overexpressed with G3BP1‐ WT or K76R mutant following CHX treatment. The results revealed that the K76R mutation in G3BP1 significantly enhances its protein stability (Figure 4P). Taken together, these results show that DCAF7 brings in USP10 to deubiquitylate G3BP1 at K76, ultimately stopping the degradation of G3BP1 through the ubiquitin‒proteasome pathway.

Additionally, to elucidate the role of the G3BP1 K76R mutant in NPC, we first silenced the endogenous G3BP1 using siRNA (si‐G3BP1) and subsequently overexpressed either the wild‐type (WT) G3BP1 or the K76R mutant (Figure S7A, Supporting Information). We conducted CCK‐8 and Transwell assays to assess the effects of the G3BP1 K76R mutation on the cisplatin sensitivity, as well as the migratory and invasive capabilities of NPC cells. Our findings demonstrated that the G3BP1 K76R mutant significantly enhanced both cisplatin resistance and the migratory and invasive abilities of NPC cells (Figure S7B,C, Supporting Information). This enhancement of oncogenic properties by the K76R mutant, which appears to surpass those of the WT G3BP1, may be attributed to its prolonged protein half‐life, thereby enabling a more sustained oncogenic activity.

### 2.7 DCAF7 Facilitates Cisplatin‐Induced Formation of SG‐Like Structures

**Figure 5:**
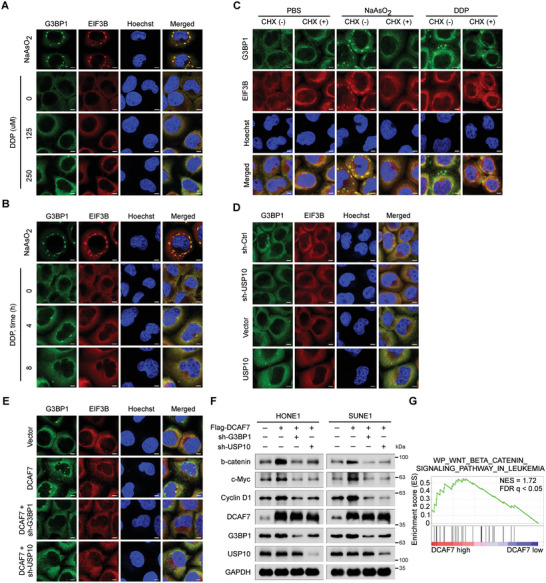
DCAF7 facilitates cisplatin‐induced formation of SG‐like structures. A,B) IF staining (with an anti‐G3BP1 or anti‐EIF3B antibody) of HONE1 cells subjected to stress induction via cisplatin (0, 125, or 250 µm) for 4 h (A) or to cisplatin treatment (250 µm) for 0, 4, or 8 h (B). As a positive control, cells were treated with 500 µm sodium arsenite (NaAsO_2_) for 1 h to induce robust SG formation. C) Cells were incubated with NaAsO_2_ (500 µm for 1 h) or cisplatin (250 µm for 4 h) and then treated with CHX (100 µg mL^−1^ for 30 min) for forced SG disassembly, and immunostaining for G3BP1 and EIF3B was then performed. D,E) IF staining (with an anti‐G3BP1 or anti‐EIF3B antibody) of HONE1 cells transfected with the indicated plasmids following cisplatin (250 µm) treatment for 4 h. The scale bar corresponds to 5 µm (A–E). F) IB of *β*‐catenin, c‐Myc, Cyclin D1, G3BP1, DCAF7, USP10 and GAPDH in HONE1 and SUNE1 cells transfected with the indicated plasmids following cisplatin treatment (10 µg mL^−1^) for 24 h. G) GSEA of the GSE102349 dataset demonstrated positive enrichment of genes associated with Wnt/*β*‐catenin signalling in response to DCAF7 overexpression. The unprocessed images of the blots are shown in Figure S10 (Supporting Information).

As G3BP1 is the pivotal determinant of SG assembly^[^ ^24^ ^]^ and USP10 plays a crucial role in G3BP1‐mediated SG assembly, we investigated whether DCAF7 and USP10 affect SG formation in NPC cells. A previous study reported the potential role of cisplatin in the formation of SG‐like structures;^[^ ^25^ ^]^ thus, we employed G3BP1 and another SG‐specific marker, EIF3B, to ascertain whether cisplatin affects SG formation in NPC cells.^[^ ^24a^ ^]^ Indeed, cisplatin induced G3BP1 foci formation in HONE1 cells in a manner that depended on concentration and time, while EIF3B did not form foci under the same conditions (**Figure** **5**A,B). Moreover, CHX inhibited the formation of SGs by preventing polysome disassembly as previously reported,^[^ ^26^ ^]^ but did not affect cisplatin induced G3BP1 foci formation, indicating that cisplatin could induce the formation of SG‐like structures rather than SGs in NPC cells (Figure 5C). To determine the contribution of USP10 to the formation of SG‐like structures under cisplatin treatment conditions, we generated USP10‐overexpressing and USP10‐knockdown NPC cells and found that overexpression of USP10 promoted cisplatin‐induced formation of SG‐like structures, whereas knockdown of USP10 suppressed this process (Figure 5D). To further demonstrate the functional role of DCAF7 and USP10 in the formation of SG‐like structures induced by cisplatin, we generated HONE1 cells using sgRNAs targeting either control (sg‐Ctrl), DCAF7 (sg‐DCAF7), or both DCAF7 and USP10 (sg‐DCAF7 plus sg‐USP10) and conducted immunofluorescence assays. Our findings indicated that the depletion of DCAF7 significantly reduced, whereas simultaneous depletion of USP10 completely abolished, the formation of cisplatin‐induced SG‐like structures (Figure S8A,B, Supporting Information). These results demonstrate that both DCAF7 and USP10 are important positive regulators in the formation of cisplatin‐induced SG‐like structures.

Given that DCAF7 promotes G3BP1 expression via USP10‐mediated deubiquitylation, we hypothesized that DCAF7 positively affects the formation of SG‐like structures. Consequently, we tracked the localization of SG‐associated constituents in DCAF7‐overexpressing NPC cells with or without G3BP1 or USP10 knockdown. As expected, overexpression of DCAF7 enhanced cisplatin‐induced formation of SG‐like structures, while this effect was abolished by G3BP1 or USP10 knockdown (Figure 5E). According to previous reports, G3BP1‐mediated formation of SGs or SG‐like structures can activate downstream signalling, including the Wnt/*β*‐catenin signalling pathway, to facilitate cancer progression.^[^ ^27^ ^]^ We then investigated whether DCAF7 affects the activation of Wnt/*β*‐catenin signalling and found that overexpression of DCAF7 enhanced the activation of this pathway. Importantly, this effect was also reversed by knockdown of either G3BP1 or USP10 (Figure 5F). In addition, GSEA of publicly available NPC RNA sequencing data revealed that elevated levels of DCAF7 resulted in significant enrichment of the Wnt/*β*‐catenin signalling pathway (Figure 5G). Collectively, the above results indicate that DCAF7 facilitates cisplatin‐induced formation of SG‐like structures and activates the Wnt/*β*‐catenin signalling pathway.

### 2.8 G3BP1 is Required for the Oncogenic Effect of DCAF7 on NPC Progression

**Figure 6:**
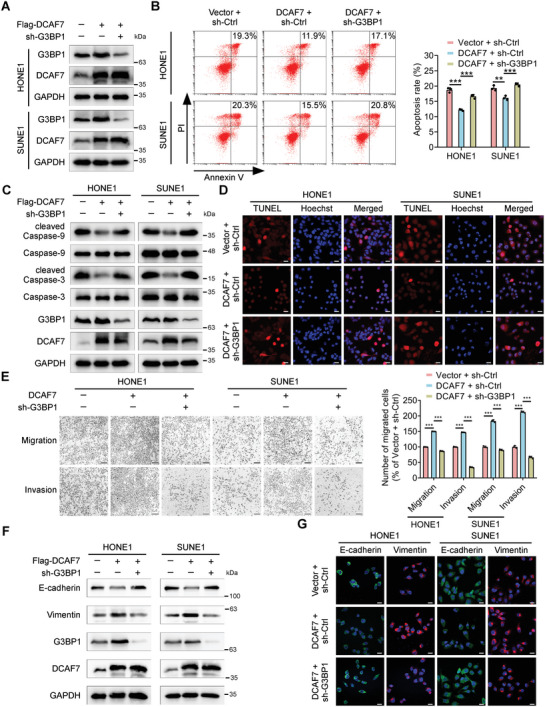
G3BP1 is required for the oncogenic effect of DCAF7 on NPC progression. A) IB of G3BP1, DCAF7 and GAPDH in HONE1 and SUNE1 cells transfected with the indicated plasmids. B) Annexin V/PI staining and flow cytometric analysis of apoptosis in HONE1 and SUNE1 cells transfected with the indicated plasmids following cisplatin treatment (2.5 µg mL^−1^) for 24 h. Mean (n = 3) ± s.d. One‐way ANOVA, ^**^ *p* < 0.01, ^***^ *p* < 0.001. C) IB of Caspase3/9, cleaved Caspase3/9, G3BP1, DCAF7 and GAPDH in HONE1 and SUNE1 cells treated with cisplatin (10 µg mL^−1^) for 24 h. D) Evaluation of apoptosis by a TUNEL assay in transfected NPC cells treated with cisplatin (10 µg mL^−1^) for 24 h. The scale bars represent 20 µm. E) Transwell assays were conducted to assess cell migration and invasion, and representative images and quantitative results are presented. The scale bars represent 200 µm. Mean (n = 3) ± s.d. One‐way ANOVA, ^***^ *p* < 0.001. F) IB of E‐cadherin, Vimentin, G3BP1, DCAF7 and GAPDH in HONE1 and SUNE1 cells transfected with the indicated plasmids. G) IF (with an anti‐E‐cadherin or anti‐Vimentin antibody) in HONE1 and SUNE1 cells transfected with the indicated plasmids. The scale bars represent 20 µm. The unprocessed images of the blots are shown in Figure S10 (Supporting Information).

To determine whether G3BP1 mediates the oncogenic function of DCAF7 in NPC, we generated stable cell lines expressing empty vector (Vector + sh‐Ctrl), DCAF7 (Flag‐DCAF7 + sh‐Ctrl) or DCAF7 plus a short hairpin RNA (shRNA) targeting G3BP1 (Flag‐DCAF7 + sh‐G3BP1) and performed in vitro functional assays in these cell lines (**Figure** **6**A). The results verified that knockdown of G3BP1 reversed the inhibition of cisplatin‐induced apoptosis mediated by ectopic expression of DCAF7 (Figure 6B). CCK‐8 assays also revealed that G3BP1 knockdown reversed the enhancement of cisplatin resistance induced by DCAF7 overexpression (Figure S9A,B, Supporting Information). In addition, the reduction in cleaved caspase 3 and caspase 9 levels mediated by ectopic expression of DCAF7 in cells treated with cisplatin were reversed by G3BP1 knockdown (Figure 6C). Further TUNEL assays revealed that the decrease in cisplatin‐induced DNA damage in DCAF7‐overexpressing cells was abolished by knockdown of G3BP1 (Figure 6D). Moreover, the enhancements in the migration and invasion capabilities of NPC cells with ectopic expression of DCAF7 were reversed by G3BP1 knockdown (Figure 6E). Consistent with these findings, G3BP1 knockdown reversed the changes in protein levels of E‐cadherin and Vimentin induced by DCAF7 overexpression, as shown by western blotting and IF staining (Figure 6F,G). Overall, these results support the hypothesis that G3BP1 serves as a functional target of DCAF7 that facilitates its oncogenic effect in NPC.

### 2.9 DCAF7 is An Independent Predictor of Unfavourable Prognosis in NPC Patients

**Figure 7:**
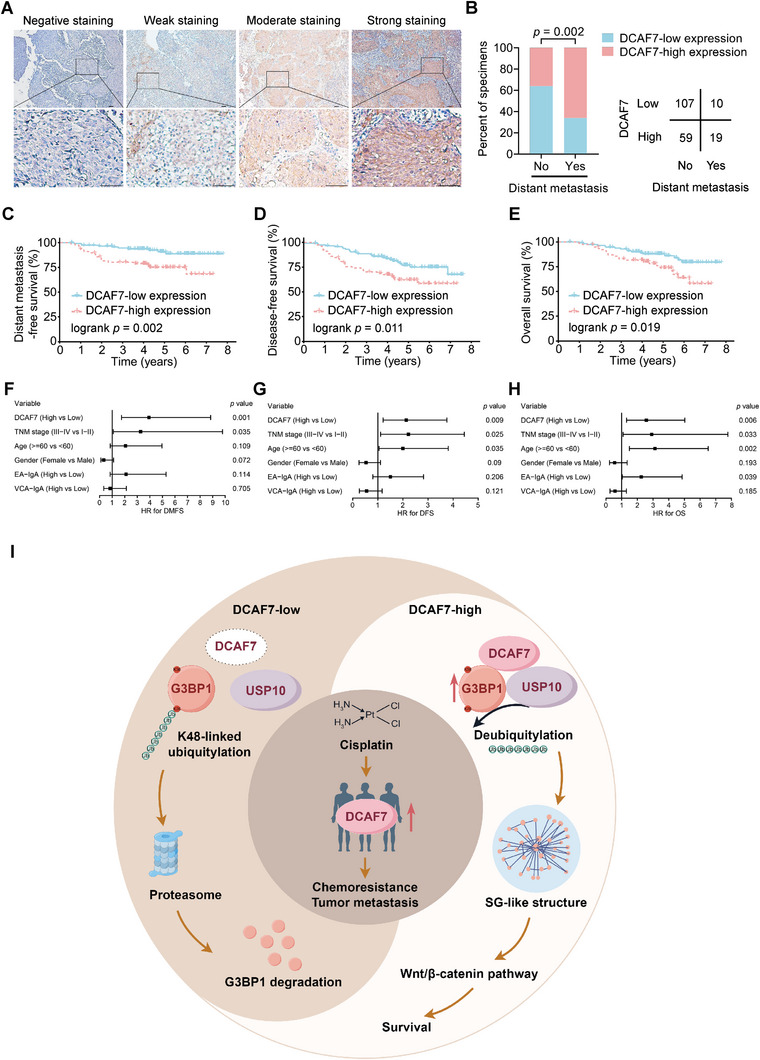
DCAF7 is an independent predictor of unfavorable prognosis in NPC patients. A) The protein expression of DCAF7 in 195 NPC tissues was scored based on the staining intensity. Scale bar = 50 µm. B) DCAF7 expression, as evaluated by IHC staining, was associated with the distant metastasis status. A two‐tailed χ2 test was used to calculate *P* values. C–E) Kaplan–Meier analysis further revealed strong correlations between DCAF7 expression and distant metastasis‐free survival (C), disease‐free survival (D), and overall survival (E), as calculated by the log‐rank test. F–H) Multivariate Cox regression analysis results revealing the prognostic significance of various clinical characteristics of NPC patients with distant metastasis‐free survival (F), disease‐free survival (G) and overall survival (H). I) Proposed working model. DCAF7 was notably upregulated in individuals with TPF‐resistant NPC, thereby enhancing cisplatin resistance and the metastatic potential of NPC cells. Mechanistically, DCAF7 functions as a scaffold to recruit USP10 for deubiquitylation and stabilization of G3BP1, thus facilitating formation of SG‐like structures in NPC cells and increasing the chemoresistance and metastasis of these cells.

To further determine the clinical relevance of DCAF7 in NPC, we conducted IHC staining with an antibody against DCAF7 in 195 NPC tissue samples. DCAF7 was expressed in both the cytoplasm and nucleolus in NPC samples. We then divided these NPC tissues into the DCAF7‐negative, DCAF7‐weak, DCAF7‐moderate, and DCAF7‐strong groups based on the DCAF7 staining intensity (**Figure** **7**A). Through comprehensive analysis of the related clinical data, we discovered a strong association between increased DCAF7 levels and an elevated risk of distant metastasis (Figure 7B; Table S2, Supporting Information). Kaplan–Meier analysis further demonstrated that increased DCAF7 levels were linked to poorer distant metastasis‐free survival, disease‐free survival and overall survival (Figure 7C–E). Furthermore, we identified DCAF7 as an independent prognostic indicator for NPC (Figure 7F–H). Our findings demonstrate that elevated levels of DCAF7 are closely linked to unfavorable outcomes in NPC patients.

## 3 Discussion

In this study, we found that DCAF7 exhibited high levels of expression in TPF‐resistant NPC patients, contributing to the cisplatin resistance and metastasis of NPC cells. Mechanistically, DCAF7 recruited USP10 for deubiquitylation and stabilization of G3BP1 and facilitated formation of SG‐like structures. Significantly, patients with NPC who had increased levels of DCAF7 expression were found to have a high risk of metastasis, leading to a negative prognosis (Figure 7I). Our study identified a novel binding partner for G3BP1 and broadened the understanding of G3BP1‐mediated formation of SG‐like structures. More importantly, we identified DCAF7 as a crucial gene in cisplatin resistance and gained insights into the mechanism behind TPF resistance in NPC patients, potentially uncovering targets for NPC treatment.

DCAF7 is a WD40‐repeat protein that can serve as a molecular scaffold for the formation of diverse multisubunit complexes.^[^ ^28^ ^]^ For example, DCAF7 facilitates complex assembly and signal transduction by binding to MEKK1 and HIPK2.^[^ ^12c^ ^]^ In addition, DCAF7 has been identified as a DDB1‐ and CUL4‐associated factor that mediates ubiquitination,^[^ ^29^ ^]^ and it has been shown to interact with DNA Ligase I and initiate its degradation via ubiquitin‐dependent pathways.^[^ ^30^ ^]^ As a scaffold protein, DCAF7 plays a critical role in sustaining protein complex stability and enabling protein–protein interactions.^[^ ^31^ ^]^ However, the specific biological function of DCAF7 in tumorigenesis and the molecular mechanisms underlying this function have not yet been fully elucidated. Our study revealed that DCAF7 functions as a scaffold to recruit USP10 for G3BP1 deubiquitylation and stabilization, a finding that expands the pool of substrates for DCAF7 as a scaffold and emphasizes the importance of ubiquitination in scaffold protein‐mediated signalling cascades.

Recently, the oncogenic role of G3BP1 has been implicated in various cancers.^[^ ^32^ ^]^ For instance, in hepatocellular carcinoma, G3BP1 promotes tumor metastasis by upregulating Slug expression.^[^ ^33^ ^]^ Conversely, depletion of G3BP1 inhibits PI3K/AKT and Wnt/*β*‐catenin signalling‐mediated proliferation and metastasis of oesophageal cancer cells.^[^ ^21b^ ^]^ Additionally, acquisition of the senescence‐associated secretory phenotype mediated by G3BP1 is a major contributor to tumor growth associated with senescence.^[^ ^34^ ^]^ However, the role of G3BP1 in NPC remains largely unexplored. Our study revealed that DCAF7 overexpression deubiquitinates G3BP1, preventing its degradation, and that G3BP1 knockdown attenuates the oncogenic effects of DCAF7 on NPC cells. The results indicate that DCAF7 targets G3BP1 in the progression of NPC. A previous study demonstrated that upon heat shock, G3BP1 undergoes K63‐linked ubiquitination, a crucial event for SG disassembly.^[^ ^35^ ^]^ However, whether the stability of G3BP1 is regulated by ubiquitination has not been determined. Here, we provide novel insights into this topic by demonstrating the degradation of G3BP1 via K48‐linked ubiquitination and the crucial involvement of the DCAF7‐USP10 axis in inhibiting the ubiquitination and degradation of G3BP1.

USP10, a ubiquitin‐specific protease, can remove ubiquitin chains from substrates and contributes to the stability of intracellular proteins.^[^ ^36^ ^]^ USP10 plays a crucial part in various cellular activities and has been implicated in the development of tumors, functioning as either an oncogene or a tumor suppressor.^[^ ^37^ ^]^ USP10 facilitates hepatocellular carcinoma proliferation by directly deubiquitinating YAP/YAZ.^[^ ^38^ ^]^ Similarly, USP10 also deubiquitinates p53 and facilitates its tumor suppressor function.^[^ ^39^ ^]^ Furthermore, knockdown of USP10 in lung cancer cells inhibits the ubiquitination of PTEN, thus promoting cell proliferation and invasion.^[^ ^40^ ^]^ Previous studies have illustrated the crucial role of USP10 in G3BP1‐mediated SG formation.^[^ ^16^ ^]^ However, whether and how USP10 can deubiquitinate G3BP1 and affect SG formation in NPC are unknown. Interference with G3BP1 has been reported to disrupt the interaction between p53 and USP10, increasing the ubiquitylation of p53.^[^ ^41^ ^]^ Moreover, G3BP1 is essential for USP10‐mediated deubiquitylation of RPS2, RPS3 and RPS10, which protect modified 40S subunits from degradation.^[^ ^23^ ^]^ Our study revealed that DCAF7 recruits USP10, which prevents the degradation of G3BP1 by eliminating its K48‐linked polyubiquitin chain at Lys76 and contributes to the formation of SG‐like structures mediated by G3BP1. These findings identify a novel binding partner for G3BP1 and broaden the understanding of G3BP1‐mediated formation of SG‐like structures, providing insight into the regulation of G3BP1 and its potential as a therapeutic target.

Overall, our study offers compelling proof that DCAF7 functions as a cisplatin resistance gene in the context of NPC progression and as a critical scaffold in G3BP1‐USP10‐mediated formation of SG‐like structures. Specifically, DCAF7 functions as a scaffold to recruit USP10 to deubiquitinate and stabilize G3BP1, thus facilitating the formation of SG‐like structures and promoting the chemoresistance and metastasis of NPC cells. Notably, DCAF7 could be an independent predictor of distant metastasis and poor prognosis in NPC patients. Our study provides novel insights into the mechanisms underlying chemoresistance in NPC and identifies potential therapeutic targets for NPC.

## 4 Experimental Section

### Clinical Specimens

Survival analysis was conducted on 195 paraffin‐embedded samples obtained from patients diagnosed with NPC at Sun Yat‐sen University Cancer Center between October 2007 and December 2009. No antitumor therapy was administered to any patient before biopsy. The staging of the tumor was conducted in accordance with the 8th version of the AJCC staging protocol. The average duration of monitoring was 54.5 months, ranging from 6.1 to 93.2 months, with the clinicopathological features of every patient detailed in Table S2 (Supporting Information). The Sun Yat‐sen University Cancer Center's Institutional Ethical Review Boards granted an exemption from the need for informed consent for this study (B2023‐073‐01).

### Cell Culture

The NPC cells along with immortalized noncancerous human nasopharyngeal epithelial NP69 cells were generously provided by Prof. Musheng Zeng (Sun Yat‐sen University Cancer Center). Human NPC cells were grown in 1640 medium (Invitrogen) with the addition of 10% fetal bovine serum (FBS; ExCell Bio, China), while NP69 cells were kept in keratinocyte/serum‐free medium (Invitrogen, Grand Island, NY, USA) with bovine pituitary extract (BD Biosciences, San Diego, CA, USA). The HEK293T cell line, sourced from the ATCC, was grown in DMEM (Invitrogen) with 10% FBS. Every cell was examined for mycoplasma infection and verified through short tandem repeat profiling. The cells were grown in culture for under 2 months.

### In Vivo Mouse Models

Five‐week‐old female BALB/c nude mice were obtained from the Guangdong Medical Experimental Animal Center. Sun Yat‐sen University Cancer Center's Experimental Animal Care and Use Committee approved the animal experiments (L025503202109031).

For the popliteal lymph node metastasis model, SUNE1 cells (2 × 10^5^) stably transduced with sh‐Ctrl or sh‐DCAF7 were injected into the footpads of mice. After 35 days, the footpad tumors and popliteal lymph nodes were collected and subjected to further analysis. Additionally, IHC staining of the popliteal lymph nodes was performed using an anti‐pancytokeratin antibody (Thermo Fisher Scientific).

In the lung metastasis experiment, mice were injected with 1 × 10^6^ SUNE1 cells that had been genetically modified to express either sh‐Ctrl or sh‐DCAF7 through the tail vein. Following an 8‐week period, the mice were sacrificed, and lung samples were gathered to assess and measure the quantity of metastatic nodules. Samples of tissue embedded in paraffin were cut into sections and then dyed using haematoxylin and eosin.

For the in vivo drug sensitivity animal assay, a total of 1 × 10^6^ stably transfected DCAF7‐knockdown or control SUNE1 cells were mixed with Matrigel (20%; BD Biosciences) and subsequently delivered subcutaneously into one dorsal flank of nude mice (n = 16 per cohort). Upon the development of discernible tumor nodules with a volume of ≈100 mm^3^, the mice in each group (sh‐Ctrl and sh‐DCAF7) were randomly divided into two subsets (each subset containing n = eight mice) and were subjected to intraperitoneal administration of either cisplatin (4 mg kg^−1^) or physiological saline solution at intervals of 3 days. The size of the tumor was observed every 3 days, and its volume was determined using the formula V = 1/2 x length x width squared. Twenty‐eight days post tumor inoculation, the mice were humanely euthanized, after which the tumors were excised and weighed. TGI was calculated using the formula: TGI (%) = (V_c_ − V_t_) / (V_c_ − V_0_) × 100, where V_c_ is the median tumor volume of the control group at the end of the study, V_t_ is the median tumor volume of the treated group, and V_0_ is the median tumor volume at the start.

### Constructs

To construct the pLKO.1‐sh‐DCAF7#1/2, sh‐USP10#1/2, and sh‐G3BP1#1/2 plasmids, shRNA sequences were inserted into the pLKO.1‐RFP vector. The shRNA sequences are shown in Table S3 (Supporting Information). The coding sequences (CDSs) of human DCAF7, USP10, and G3BP1 were separately cloned into the pSin‐EF2‐puro vector. The PRK‐HA‐Ub plasmid was obtained as previously described.^[^ ^42^ ^]^ Lipofectamine 3000 reagent (Invitrogen) was utilized for plasmid transfection. For the generation of NPC cells with stable knockdown of DCAF7, lentiviral vectors harbouring shRNA targeting DCAF7 or a scrambled control shRNA were designed and subsequently synthesized. The SUNE1 and HONE1 cells were dispensed into six‐well culture plates (NEST Biotechnology) prior to viral transduction at predetermined titres. The cells were subsequently subjected to puromycin selection (1 µg mL^−1^) for 1 week. Validation was then carried out using reverse transcription–quantitative PCR (RT‒qPCR) and western blotting.

### RNA Isolation and RT‒qPCR

Cells were used to extract total RNA with the TRIzol reagent (Invitrogen). The initial cDNA strand was created with the help of M‐MLV reverse transcriptase from Promega and random primers. The amplification of cDNA was performed using platinum SYBR Green qPCR Super Mix‐UDG reagents (Invitrogen) on a CFX96 Touch sequence detection system from Bio‐Rad. Tubulin was used as a reference control for all the genes. The 2^−ΔΔCT^ method was utilized to determine relative gene expression. Table S3 (Supporting Information) contains the list of primers utilized in the sequences.

### Western Blotting

Lysis of cells was performed on ice using RIPA buffer with a protease inhibitor cocktail from Fdbio Science in Hangzhou, China. The lysates' proteins were isolated using 7.5–15% SDS‒PAGE gels and subsequently moved to PVDF membranes from Merck Millipore in Billerica, MA, USA. Following blocking of the membranes with 5% nonfat milk, primary antibodies against various proteins including DCAF7 (1:1000; Abcam, ab138490), G3BP1 (1:1000; Abcam, ab181150), USP10 (1:1000; Proteintech, 19374‐1‐AP), Caspase‐3 (1:1000; CST, 14220), cleaved Caspase‐3 (1:1000; Abcam, ab32042), Caspase‐9 (1:1000; CST, 9508), cleaved Caspase‐9 (1:1000; CST, 7237), E‐cadherin (1:1000; Proteintech, 60335‐1‐Ig), Vimentin (1:1000; Proteintech, 10366‐1‐AP), *β*‐catenin (1:1000; Proteintech, 67447‐1‐Ig), c‐Myc (1:1000; Proteintech, 67447‐1‐Ig), Cyclin D1 (1:1000; Proteintech, 60186‐1‐Ig), Flag (1:1000; Sigma, F1804), HA (1:1000; Sigma, H6908), Myc‐tag (1:1000; Proteintech, 16286‐1‐AP), α‐tubulin (1:1000; Proteintech, 66031‐1‐Ig), and GAPDH (1:5000; Abcam, ab128915) were incubated overnight at 4 °C. ECL detection system from Thermo Fisher Scientific was used to detect signals following incubation with secondary antibodies.

### IF Staining

Cells transfected with plasmids were placed on glass coverslips and treated with methanol after 24 h. Following this, the cells were left to incubate overnight at 4 °C with primary antibodies targeting E‐cadherin (1:100; Proteintech, 60335‐1‐Ig), Vimentin (1:100; Proteintech, 10366‐1‐AP), DCAF7 (1:100; Abcam, ab138490), G3BP1 (1:100; BD Biosciences, 611126) and EIF3B (1:100; Proteintech, 10319‐1‐AP). Afterwards, the cells were cultured with Alexa Fluor 594‐ or 488‐labeled IgG as a follow‐up antibody (1:1000; Life Technologies, A21207 and A21202). Cells were exposed to a TUNEL reaction mixture containing terminal deoxynucleotidyl transferase, nucleotides, and YF594‐labelled dUTP for the TUNEL assay. After counterstaining the cells with Hoechst 33342, images were captured with a confocal laser scanning microscope from Olympus FV1000 in Tokyo, Japan.

### Transwell Assay

Either 5 × 10^4^ cells (for the migration test) or 1 × 10^5^ cells (for the invasion test) were placed in 200 µL of serum‐free medium in Transwell chambers (8 µm pores; Corning, NY, USA). The membrane was either precoated with Matrigel (for the invasion test) or left uncoated (for the migration test). The lower chambers were filled with additional medium. After incubation for indicated time (12 h for HONE1 cells and 16 h for SUNE1 cells in the migration test; 20 h for HONE1 cells and 24 h for SUNE1 cells in the invasion test), the cells were fixed and stained, and the invaded or migrated NPC cells were counted.

### Cell Viability Assay

HONE1 and SUNE1 cells were placed in 96‐well plates at a concentration of 2 to 3 × 10^3^ cells per well for the drug sensitivity test conducted in vitro. Afterward, the cells were treated with cisplatin at different doses (ranging from 0.15 to 10 µg mL^−1^) for 48 h. Subsequently, the cells were cultured for an extra 2 h with CCK‐8 reagent from TargetMol. Cell viability was assessed by determining the absorbance at a wavelength of 450 nm.

### Flow Cytometric Apoptosis Assay

For the apoptosis assay, cells were treated with cisplatin (2.5 µg mL^−1^). Following a 48‐h incubation period, the cells were harvested, rinsed with PBS, and then suspended in 500 µL of 1 × binding buffer. The suspension was then left to incubate in the dark at room temperature for 15 min with 5 µL of Annexin V/FITC and 5 µL of propidium iodide (PI). The apoptosis rate was determined using a cytoFLEX flow cytometer and analyzed with CytExpert 2.2 software. Both FITC+/PI‐ and FITC+/PI+ cells were identified as apoptotic cells.

### GSEA

The gene expression profiles of 113 NPC specimens (GSE102349) were examined to identify variations in gene expression levels among patients with differing levels of DCAF7 expression. Using the curated gene set collection from the Molecular Signatures Database C2, genes associated with either high or low expression of DCAF7 were assessed. The GSEA results were presented as normalized enrichment scores. A false discovery rate (FDR) of < 0.25 and *P* < 0.05 were set as the thresholds for determining statistical significance.

### Co‐IP and MS

Cells were lysed using Pierce IP Lysis Buffer (Thermo Fisher Scientific) with the addition of protease inhibitor cocktail. The lysates underwent immunoprecipitation using either an anti‐FLAG antibody (3 µg; Sigma, F1804) or IgG (3 µg; Invitrogen, 10500C) for a duration of overnight at 4 °C. Protein A/G magnetic beads (Thermo Scientific) were added to the precipitated immune complexes. The immune complexes that were washed out were then analyzed using western blot or MS as detailed in a previous study.^[^ ^43^ ^]^

### Denaturing IP Assay

The ubiquitination test was performed in denaturing circumstances as outlined before.^[^ ^44^ ^]^ NPC cells were transfected and then incubated with 10 µm MG132 for 6 h after 24 h, followed by lysis in a buffer with EDTA‐free protease inhibitor cocktail from Roche. Following lysis using 100 µL of buffer, the lysates were heated at 95 °C for 5 min with 1% SDS to denature. The denatured lysates were diluted with lysis buffer to an SDS concentration of less than 0.1% and were then subjected to immunoprecipitation with the indicated antibodies. The level of ubiquitination was measured using Co‐IP with an anti‐Flag antibody and western blotting with an anti‐HA antibody.

### IHC Staining and Scoring

Immunohistochemical staining and evaluation were conducted following the methods described in a previous study.^[^ ^45^ ^]^ Briefly, sections of NPC tissue embedded in paraffin were treated to remove the paraffin and restore hydration, followed by the suppression of natural peroxidase activity. Following the prevention of non‐specific protein binding, the slides were left to incubate with primary antibodies overnight at a temperature of 4 °C. The primary antibodies were subsequently labelled with a horseradish peroxidase (HRP)‐conjugated secondary antibody. Haematoxylin was employed to stain the cell nuclei. Visualization and analysis of IHC staining were performed with the AxioVision Rel.4.6 computerized image analysis system (Carl Zeiss). Two pathologists independently assessed the level of staining. Staining intensity was assessed from 0 (absence of staining) to 3 (intense staining), while the percentage of positive cells was evaluated from 1 (< 10%) to 4 (>70%). To determine the overall staining score for each specified protein, the scores for staining intensity and the proportion of positive cells were multiplied together.

To analyze the correlation between G3BP1 and DCAF7 expression in lung metastatic nodules of a mouse model, lung tissue sections from the model mice were stained using IHC as previously outlined.

### Differential Expression and Prognostic Analysis of DCAF7 in Pan‐Cancer

The uniformly standardized pan‐cancer dataset was sourced from the UCSC database (https://xenabrowser.net/). Within this dataset, expression data for DCAF7 across multiple samples was extracted. Utilizing R software (version 3.6.4), the differential expression between normal and tumor tissues for each type of cancer was quantified using unpaired Wilcoxon Rank Sum and Signed Rank Tests to determine statistically significant disparities. Additionally, a Kaplan–Meier survival curve was generated via the Kaplan–Meier Plotter, selecting the optimal expression cut‐off for demarcation (http://kmplot.com/analysis/).

### Analysis of Protein–Protein Interactions

The protein–protein interaction analysis was conducted using the STRING database (http://string‐db.org/) with a confidence threshold set at 0.4, indicative of medium confidence.^[^ ^46^ ^]^ To delineate sub‐networks of functionally correlated genes, the Molecular Complex Detection (MCODE) algorithm integrated into the Cytoscape software, was employed, utilizing default parameters.^[^ ^47^ ^]^

### Statistical Analysis

The mean and standard deviation (s.d.) data was collected from a minimum of three separate trials. Student's *t*‐test, one‐way ANOVA, or two‐way ANOVA were used to compare continuous variables, while Fisher's exact test was used to compare categorical variables. Receiver operating characteristic (ROC) curve analysis was used to establish the ideal threshold for DCAF7 expression. Survival curves were plotted using the Kaplan‒Meier technique, with corresponding P values calculated through the log‐rank test. Independent prognostic factors were identified using a multivariate Cox proportional hazards regression model. A significance level of less than 0.05 was deemed to be statistically significant. The data underwent statistical analysis with SPSS software version 22.0 (IBM, Chicago, IL, USA) and R statistical software version 4.2.0.

## Conflict of Interest

The authors declare no conflict of interest.

## Author Contributions

Q.J.L., X.L.F., Y.Q.L., and J.Y.L. contributed equally to this work. L.L.T., Y.Z., and Q.J.L. conceived the study and designed the experiments. Q.J.L. and X.L.F., carried out most of the experiments and analyzed the data. Y.Q.L., J.Y.L., C.L.H., S.W.H., S.Y.H., J.Y.L., and S.G. helped with the experiments and provided technical assistance. N.L. and J.M. provided equipment. L.L.T. and Y.Z. provided funding support and regents. L.L.T., Y.Z., and Q.J.L. wrote and revised the manuscript. All authors reviewed and approved the final manuscript.

## Supporting information

Supporting Information

## Data Availability

The key raw data were uploaded to the Research Data Deposit public platform (http://www.researchdata.org.cn, RDDB2024312357) and are available from the corresponding author upon reasonable request.
