# Supplementary material for: DCAF7 Acts as A Scaffold to Recruit USP10 for G3BP1 Deubiquitylation and Facilitates Chemoresistance and Metastasis in Nasopharyngeal Carcinoma
Source: Adv Sci (Weinh). 2024 Jul 8;11(36):2403262. doi: 10.1002/advs.202403262 (PMC11423104; doi:10.1002/advs.202403262)
Supplement: Supplementary file 1 — Supporting Information [file ADVS-11-2403262-s001.pdf]

ADVANCED SCIENCE
Open Access

Supporting Information

for *Adv. Sci.*, DOI 10.1002/advs.202403262

DCAF7 Acts as A Scaffold to Recruit USP10 for G3BP1 Deubiquitylation and Facilitates Chemoresistance and Metastasis in Nasopharyngeal Carcinoma

Qing-Jie Li, Xue-Liang Fang, Ying-Qin Li, Jia-Yi Lin, Cheng-Long Huang, Shi-Wei He, Sheng-Yan Huang, Jun-Yan Li, Sha Gong, Na Liu, Jun Ma, Yin Zhao and Ling-Long Tang**

Supporting information

DCAF7 Acts as A Scaffold to Recruit USP10 for G3BP1 Deubiquitylation and Facilitates Chemoresistance and Metastasis in Nasopharyngeal Carcinoma

Qing-Jie Li, Xue-Liang Fang, Ying-Qin Li, Jia-Yi Lin, Cheng-Long Huang, Shi-Wei He, Sheng-Yan Huang, Jun-Yan Li, Sha Gong, Na Liu, Jun Ma, Yin Zhao, Ling-Long Tang**

Supplementary figures

Figure S1. DCAF7 is highly expressed in most of solid tumors and associated with poor prognosis

Figure S2. Overexpression of DCAF7 facilitates the cisplatin resistance of NPC cells

Figure S3. DCAF7 enhances NPC cell migration, invasion and EMT

Figure S4. Identification of hub proteins from the PPI network with the MCODE algorithm

Figure S5. Overexpression of DCAF7 prolongs the half-life of G3BP1

Figure S6. Overexpression of USP10 prolongs the half-life of G3BP1

Figure S7. Significant enhancement of cisplatin resistance and migratory and invasive capabilities in NPC cells expressing the G3BP1 K76R mutant

Figure S8. Both DCAF7 and USP10 are important positive regulators in the formation of cisplatin-induced SG-like structures

Figure S9. DCAF7 promotes NPC cisplatin resistance via G3BP1

Figure S10. Unprocessed immunoblots for indicated Figures panels

Supplementary tables

Table S1. Mass spectrometry analysis results for the anti-FLAG (FLAG-tagged DCAF7) immunoprecipitation complex

Table S2. Correlations between DCAF7 expression levels and clinical features in Nasopharyngeal carcinoma patients

Table S3 List of primers used in this study

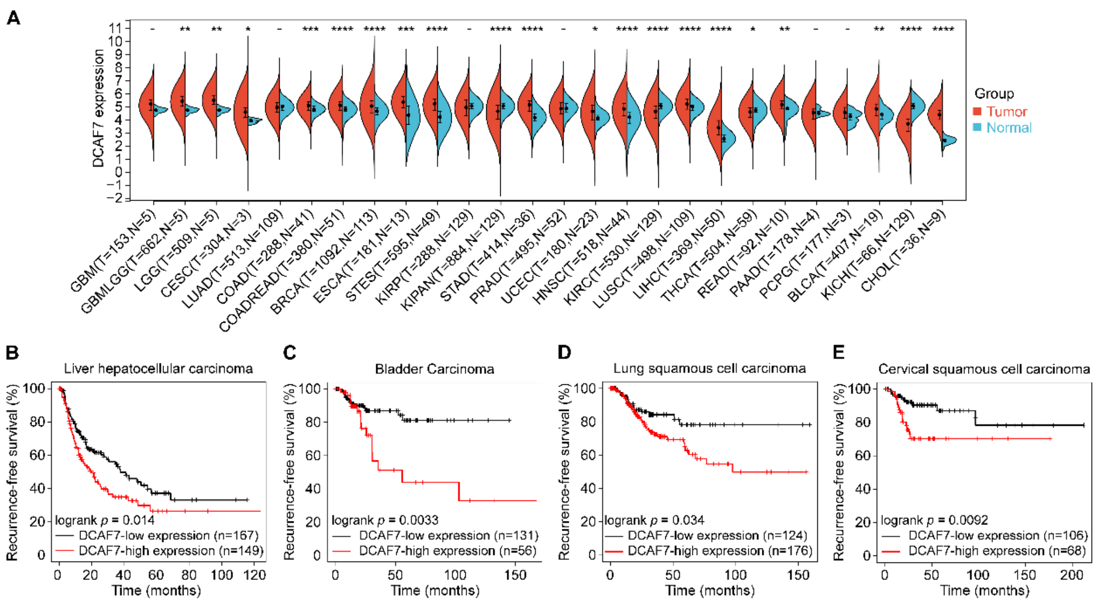

Figure S1. DCAF7 is highly expressed in most of solid tumors and associated with poor prognosis. A) The mRNA expression of DCAF7 in tumor and normal tissue samples was examined by analyzing TCGA database. GBM, glioblastoma multiforme; GBMLGG, glioma; LGG, brain lower grade glioma; CESC, cervical squamous cell carcinoma and endocervical adenocarcinoma; LUAD, lung adenocarcinoma; COAD, colon adenocarcinoma; COADREAD, colon adenocarcinoma/rectum adenocarcinoma esophageal carcinoma; BRCA, breast invasive carcinoma; ESCA, esophageal carcinoma; STES, stomach and esophageal carcinoma; KIRP, kidney renal papillary cell carcinoma; KIPAN, pan-kidney cohort (kidney chromophobe + kidney renal clear cell carcinoma + kidney renal papillary cell carcinoma); STAD, stomach adenocarcinoma; PRAD, prostate adenocarcinoma; UCEC, uterine corpus endometrial carcinoma; HNSC, head and neck squamous cell carcinoma; KIRC, kidney renal clear cell carcinoma; LUSC, lung squamous cell carcinoma; LIHC, liver hepatocellular carcinoma; THCA, thyroid carcinoma; READ, rectum adenocarcinoma; PAAD, pancreatic adenocarcinoma; PCPG, pheochromocytoma and paraganglioma; BLCA, bladder urothelial carcinoma; KICH, kidney chromophobe; CHOL, cholangiocarcinoma. Unpaired Wilcoxon Rank Sum and Signed Rank Tests were used, *$P < 0.05$, **$P < 0.01$, ***$P < 0.001$, and ****$P < 0.0001$. B-E) Survival analysis of DCAF7 in TCGA dataset.

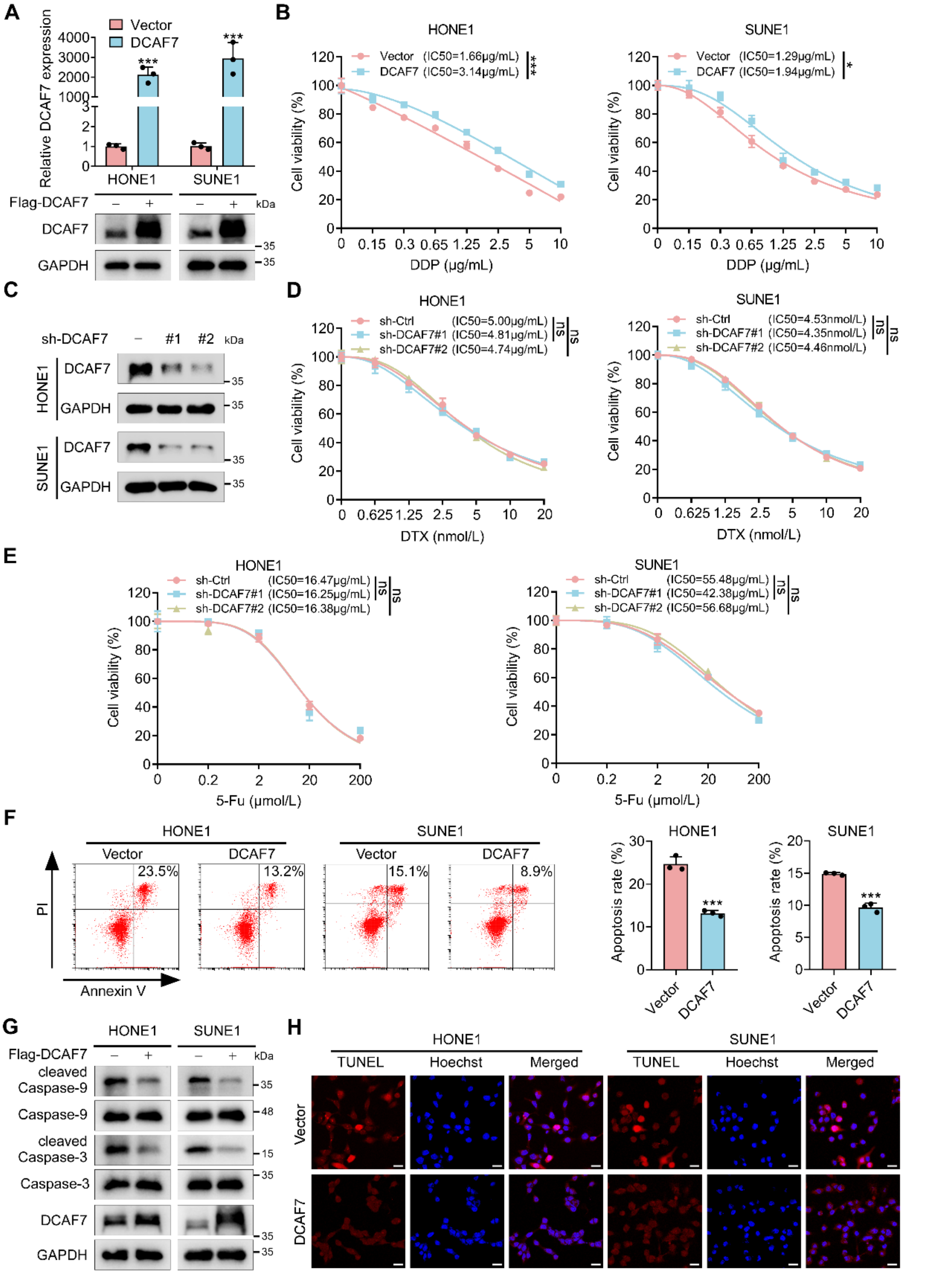

Figure S2. Overexpression of DCAF7 facilitates the cisplatin resistance of NPC cells. A) The efficiency of DCAF7 overexpression was assessed using RT-qPCR and western blotting assays in HONE1 and SUNE1 cells. Mean (n = 3) ± s.d. Student's t-test, *** *P* < 0.001. B) HONE1 or SUNE1 cells were subjected to indicated doses of cisplatin for 48 h, and the NPC cell viability was evaluated using CCK-8. Mean (n = 4) ± s.d. Two-

way ANOVA, * $P < 0.05$, *** $P < 0.001$. C) The DCAF7 knockdown efficiency was assessed using western blotting. D, E) A CCK-8 assay was used to evaluate docetaxel (D) and 5-fluorouracil (E) resistance in transfected NPC cells following treatment with the indicated concentrations of cisplatin for 48 h. Mean (n = 4) ± s.d. Two-way ANOVA, ns $P > 0.05$. F) HONE1 or SUNE1 cells with DCAF7-overexpression were treated with cisplatin (2.5 μg/mL) for 24 h, and Annexin-V/PI analysis was conducted using flow cytometry. Mean (n = 3) ± s.d. Student's t-test, *** $P < 0.001$. G) Western blotting assays (anti-cleaved Caspase3/9, Caspase3/9, anti-GAPDH or anti-DCAF7) in DCAF7-overexpressing HONE1 and SUNE1 cells following treatment with cisplatin (10 μg/mL) for 24 h. H) HONE1 or SUNE1 cells were treated with 10 μg/mL cisplatin for 24 h and then subjected to TUNEL staining. Scale bars = 20 μm. The unprocessed images of the blots are shown in Figure S10.

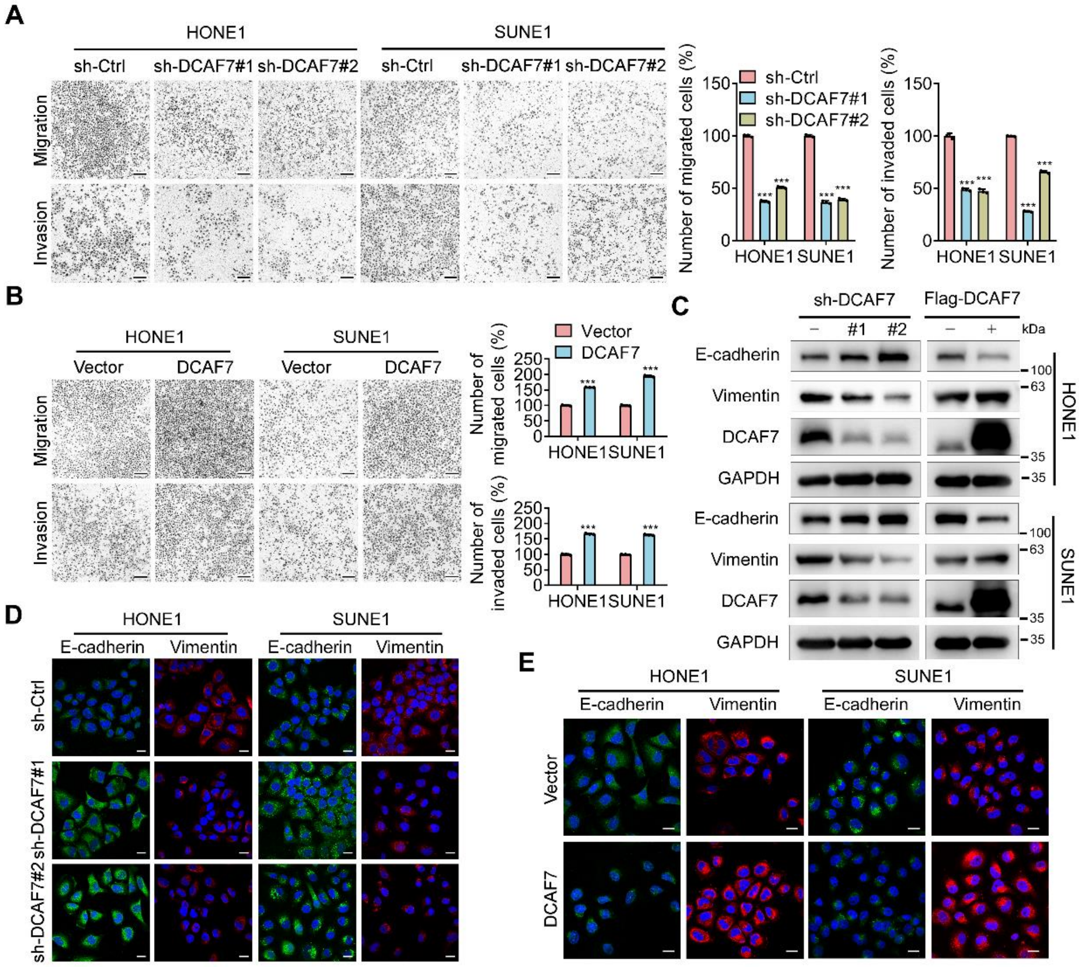

Figure S3. DCAF7 enhances NPC cell migration, invasion and EMT. A, B) Transwell assays were used to examine the impact of DCAF7 knockdown or overexpression on the migration and invasion of HONE1 and SUNE1 cells. The scale bars were set to 200 μm. Mean (n = 3) ± s.d. One-way ANOVA in A and Student's t-test in B were used, *** $P < 0.001$. C-E) E-cadherin and Vimentin levels were evaluated by western blotting (anti-GAPDH, anti-DCAF7, anti-Vimentin or anti-E-cadherin) and immunofluorescence staining (anti-Vimentin and anti-E-cadherin) in HONE1 and SUNE1 cells after DCAF7 overexpression or knockdown. The scale bars were set to 20 μm. The unprocessed images of the blots are shown in Figure S10.

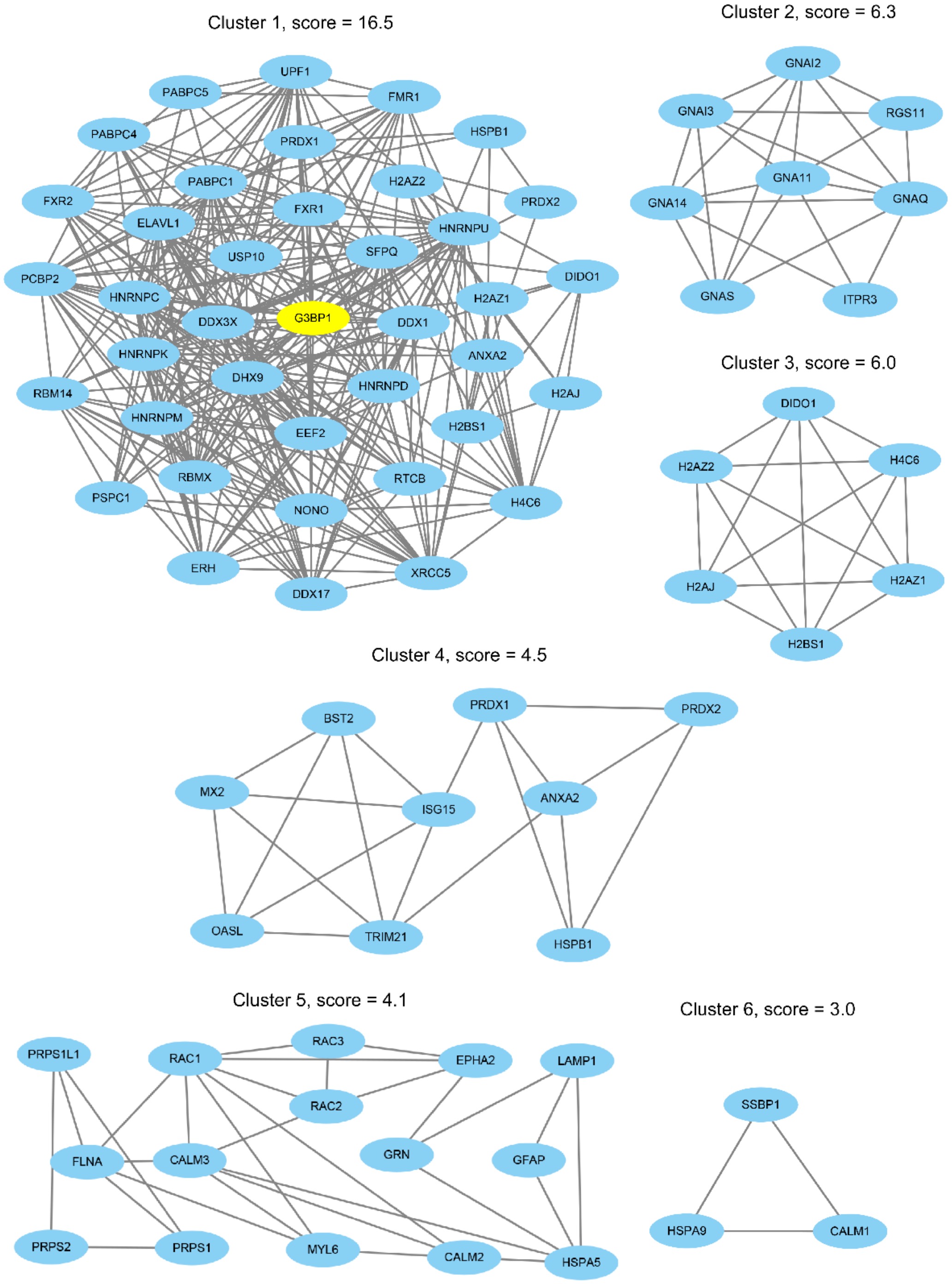

Figure S4. Identification of hub proteins from the PPI network with the MCODE algorithm. Substantial gene clusters were extracted by considering the score of MCODE and the number of interconnected nodes. The clusters were inferred using default settings, including a degree cutoff of 2, a node score cutoff of 0.2, a K core of 2, and a maximum depth of 100.

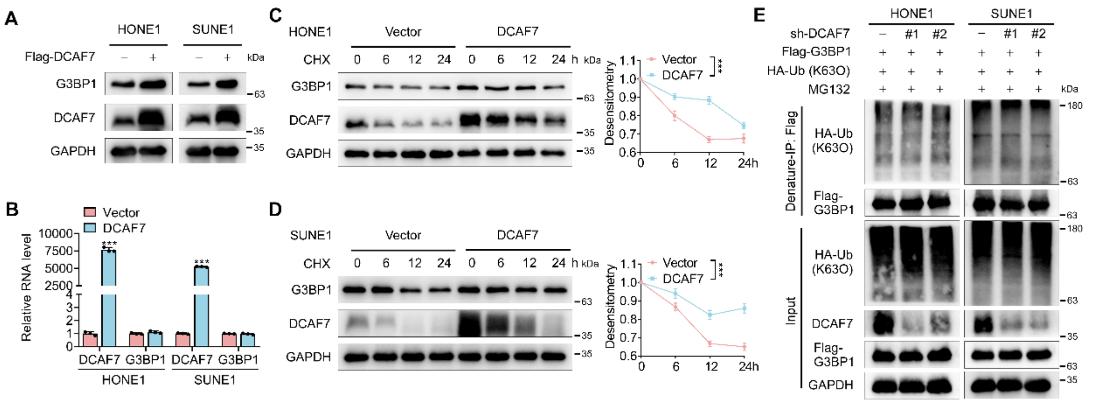

Figure S5. Overexpression of DCAF7 prolongs the half-life of G3BP1. A, B) G3BP1 expression in DCAF7-overexpressing and control HONE1 or SUNE1 cells was evaluated through western blotting and RT-qPCR assays. Mean (n = 3) ± s.d. Student's t-test, *** $P < 0.001$. C, D) G3BP1, DCAF7 and GAPDH protein levels in HONE1 and SUNE1 cells following CHX treatment (100 μg/mL) for indicated times. Mean (n = 3) ± s.d. Two-way ANOVA, *** $P < 0.001$. E) Denaturing IP (with an anti-Flag antibody) and IB of HA, Flag, DCAF7 and GAPDH in HONE1 and SUNE1 cells transfected with the indicated plasmids following MG132 treatment (10 μM, 6 h). The unprocessed images of the blots are shown in Figure S10.

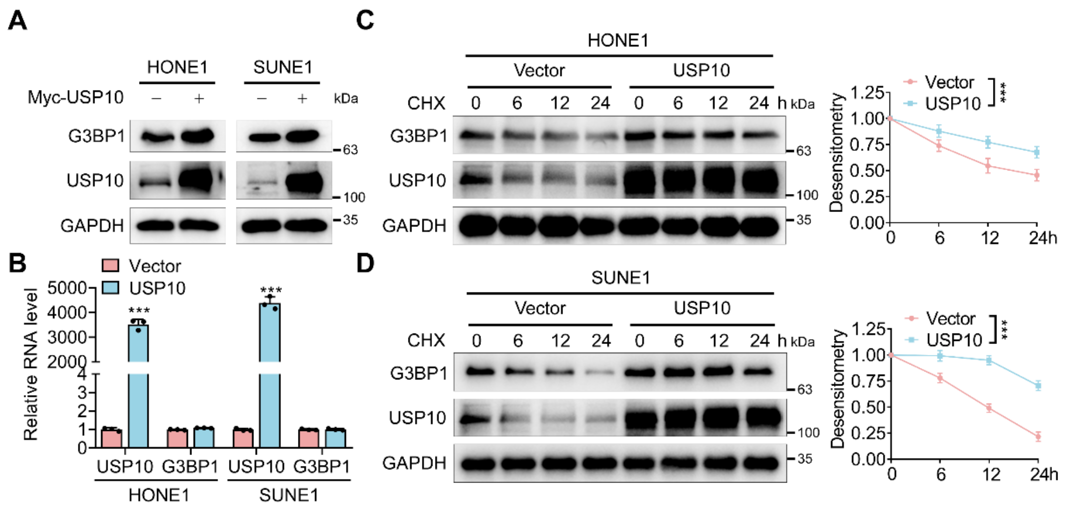

Figure S6. Overexpression of USP10 prolongs the half-life of G3BP1. A, B) G3BP1 expression in USP10-overexpressing and control HONE1 or SUNE1 cells was evaluated through western blotting and RT-qPCR assays. Mean (n = 3) ± s.d. Student's t-test, *** $P < 0.001$. C, D) G3BP1, USP10 and GAPDH protein levels in HONE1 and SUNE1 cells following CHX treatment (100 µg/mL) for indicated times. Mean (n = 3) ± s.d. Two-way ANOVA, *** $P < 0.001$. The unprocessed images of the blots are shown in Figure S10.

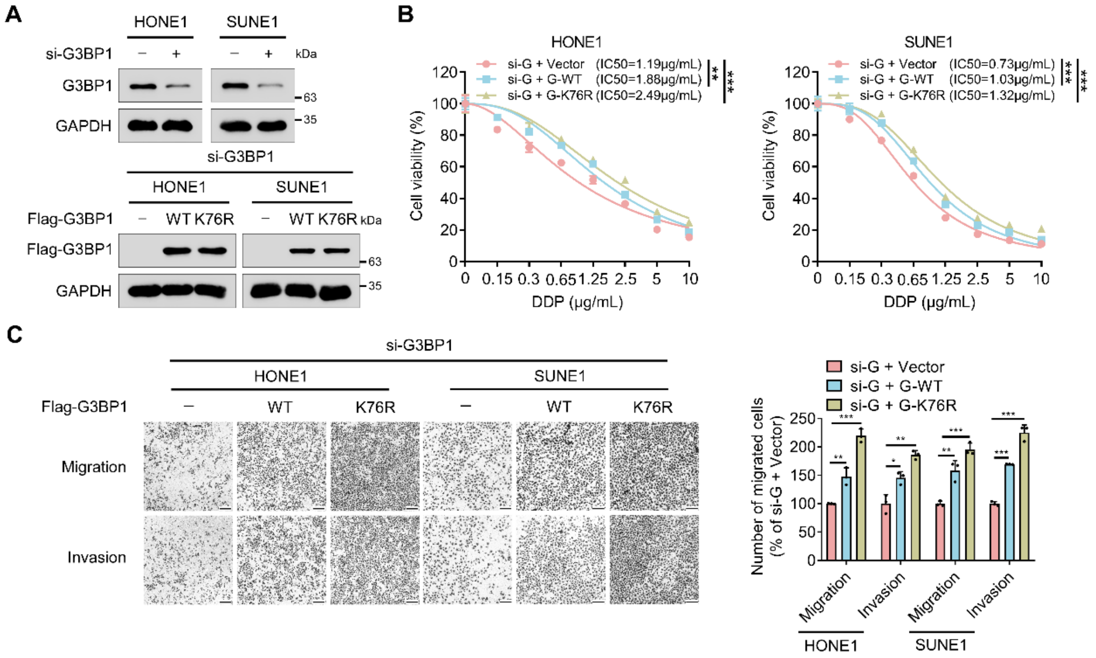

Figure S7. Significant enhancement of cisplatin resistance and migratory and invasive capabilities in NPC cells expressing the G3BP1 K76R mutant. A) Protein levels of G3BP1, Flag-G3BP1 and GAPDH in HONE1 and SUNE1 cells transfected with siRNA targeting G3BP1 (si-G3BP1) and either empty vector, Flag-G3BP1 WT, or K76R. B) A CCK-8 assay evaluating cisplatin resistance in NPC cells transfected with si-G3BP1, Flag-G3BP1 WT, or K76R following treatment with specified concentrations of cisplatin for 48 hours. Data are presented as mean (n = 4) ± s.d. Statistical significance was determined using a two-way ANOVA, ** $P < 0.01$. C) Transwell assays were conducted in HONE1 and SUNE1 cells transfected with siRNA targeting G3BP1 (si-G3BP1) and either empty vector, Flag-G3BP1 WT, or K76R. Representative images and quantitative results are presented, with scale bars representing 200 μm. Mean (n = 3) ± s.d. One-way ANOVA, * $P < 0.05$, ** $P < 0.01$, *** $P < 0.001$. The unprocessed images of the blots are shown in Figure S10.

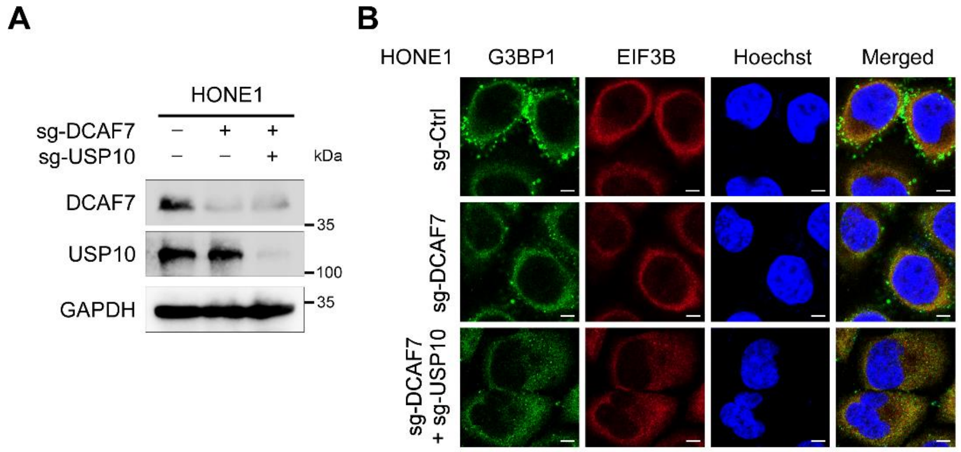

Figure S8. Both DCAF7 and USP10 are important positive regulators in the formation of cisplatin-induced SG-like structures. A) The efficiency of DCAF7 and USP10 knockout was confirmed via western blot analysis. B) Immunofluorescence staining (using anti-G3BP1 or anti-EIF3B antibodies) of HONE1 cells transfected with the indicated sgRNAs following treatment with cisplatin (250 μM) for 4 hours. Scale bar represents 5 μm. The unprocessed images of the blots are shown in Figure S10.

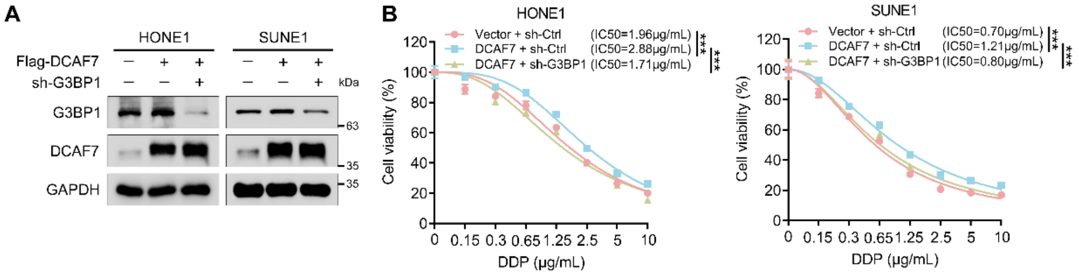

Figure S9. DCAF7 promotes NPC cisplatin resistance via G3BP1. A) Western blot analysis was performed to assess the efficiency of DCAF7 overexpression and G3BP1 knockdown. B) A CCK-8 assay was used to evaluate cisplatin resistance in NPC cells transfected with the indicated plasmids following treatment with various concentrations of cisplatin for 48 h. Mean (n = 4) ± s.d. Two-way ANOVA, ** $P < 0.01$. The unprocessed images of the blots are shown in Figure S10.

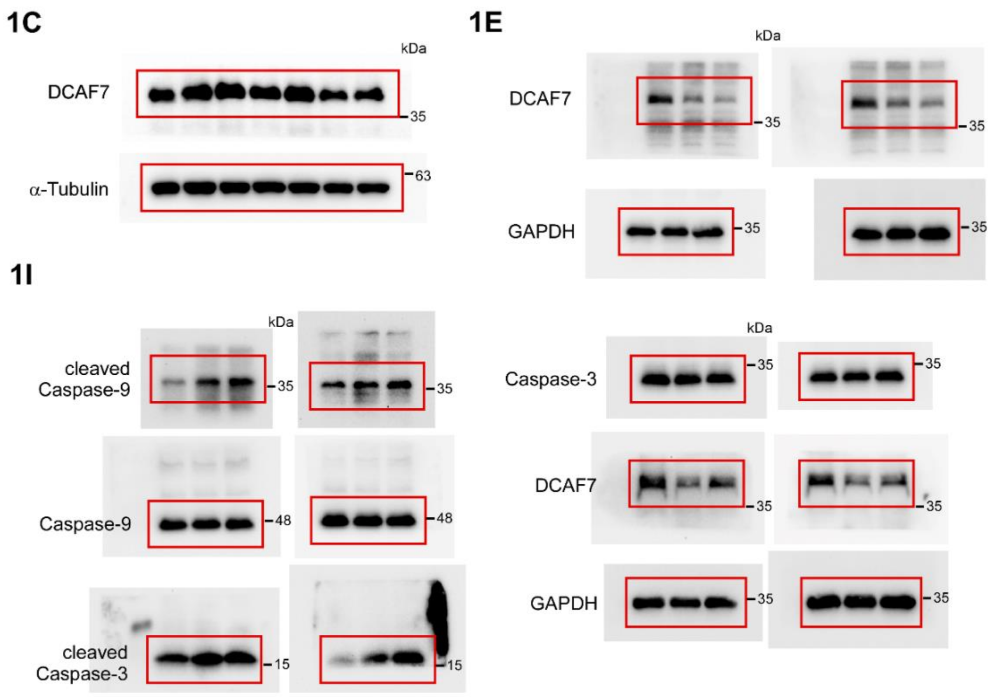

Figure S10. (Unprocessed immunoblots**)**

Unprocessed immunoblots for indicated Figures panels.

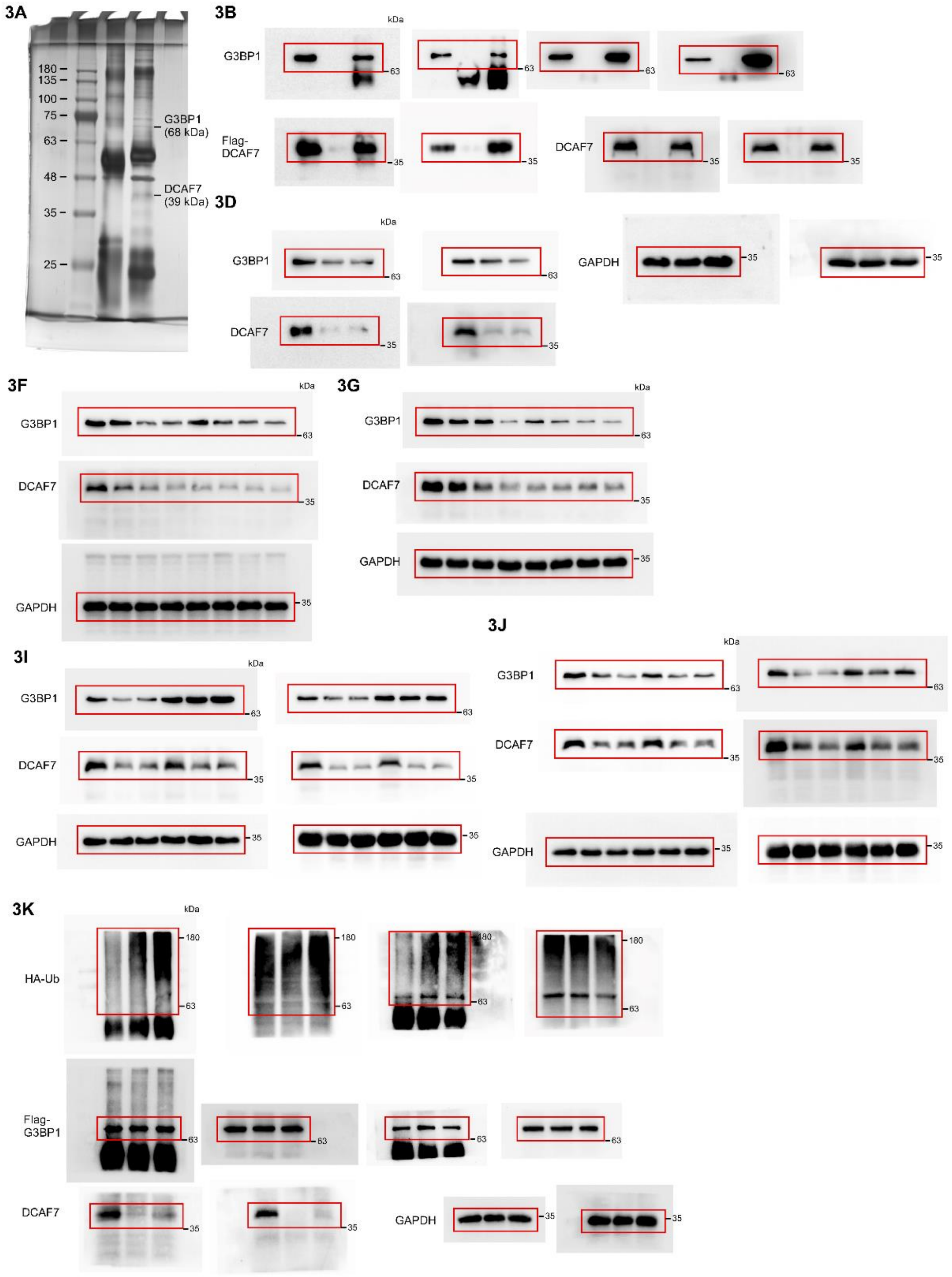

Figure S10. (cont'd) **(**Unprocessed immunoblots (continued)**)**

Unprocessed immunoblots for indicated Figures panels.

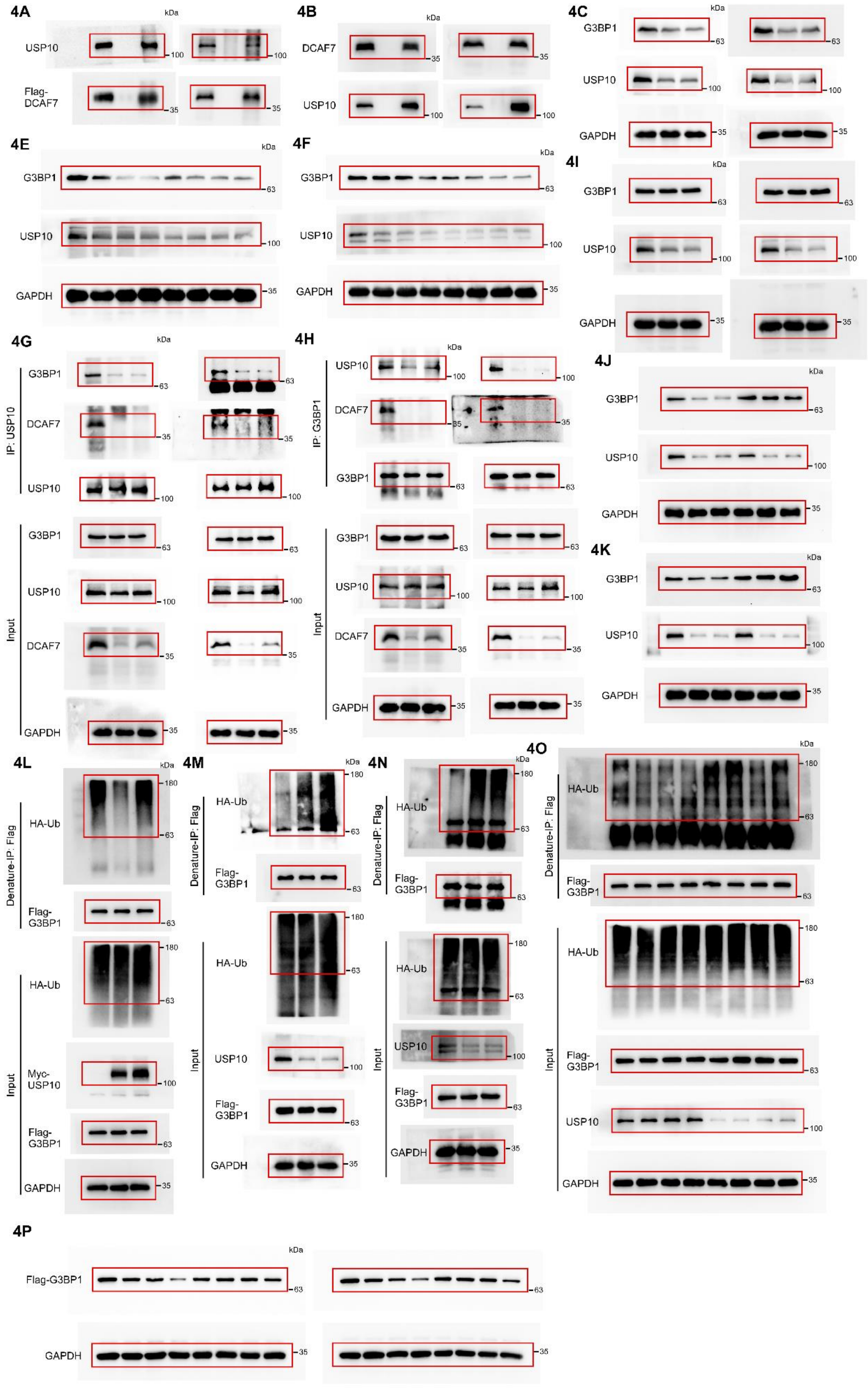

Figure S10. (cont'd) (Unprocessed immunoblots (continued))

Unprocessed immunoblots for indicated Figures panels.

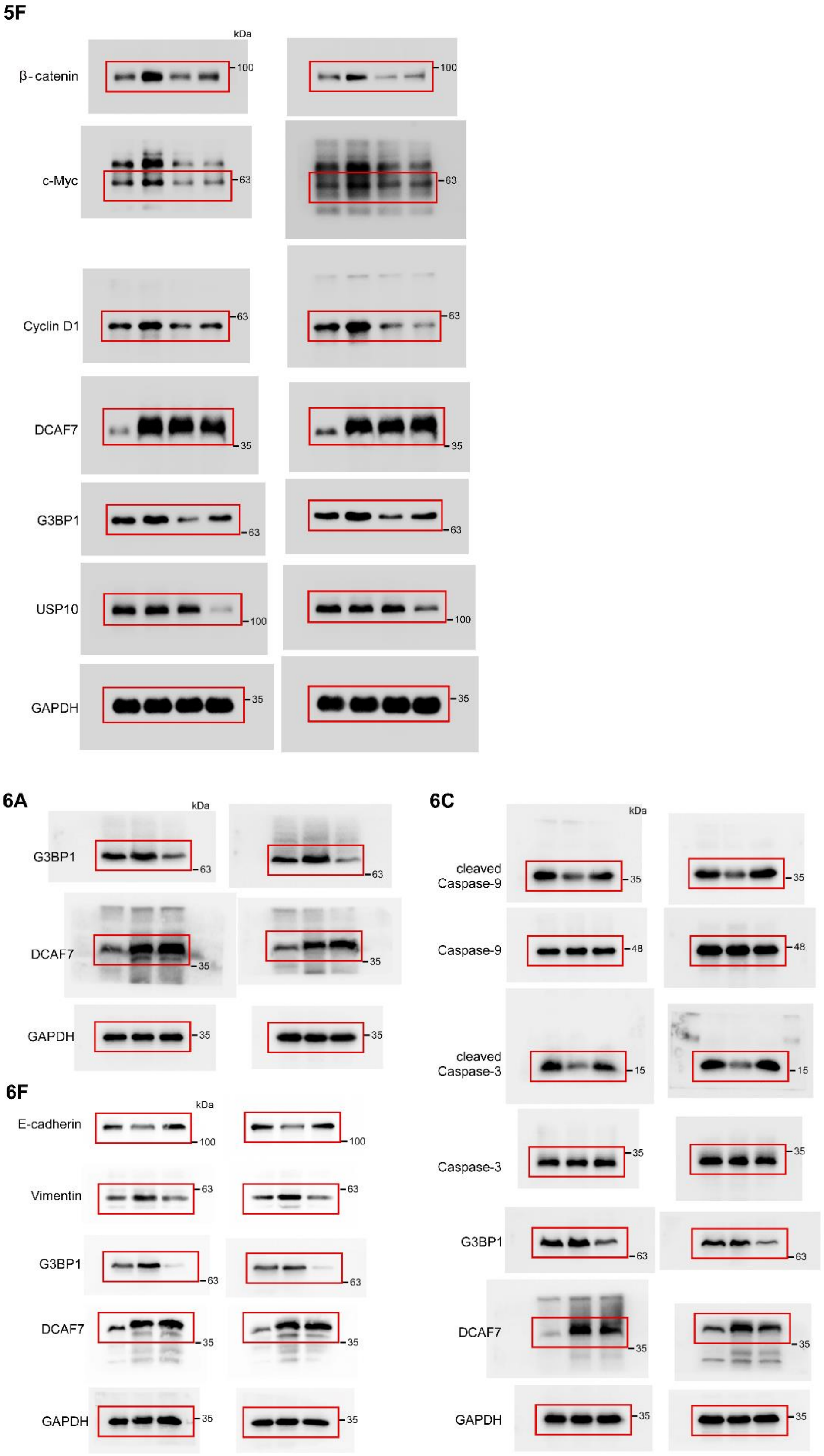

Figure S10. (cont'd) (Unprocessed immunoblots (continued)**)**

Unprocessed immunoblots for indicated Figures panels.

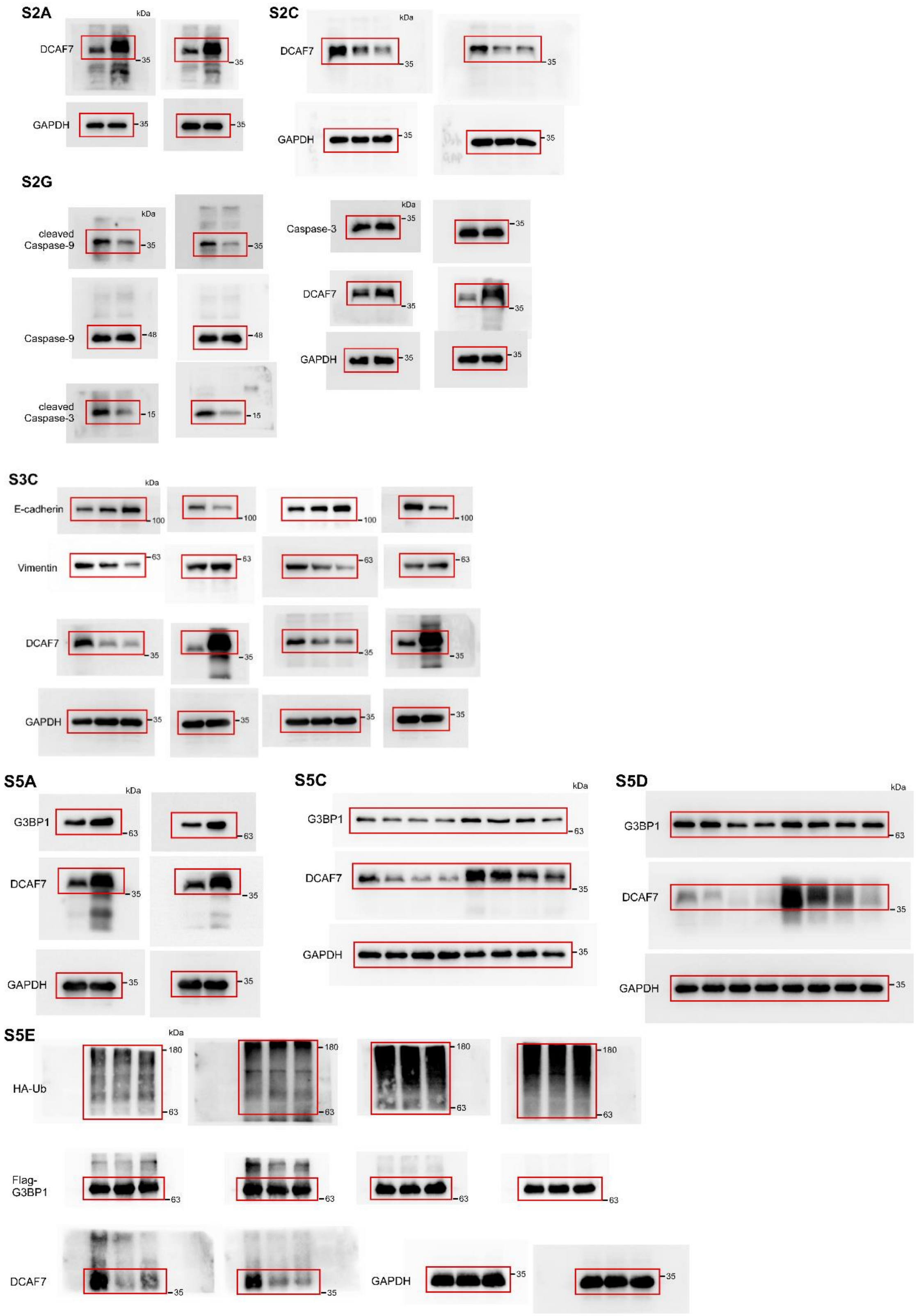

Figure S10. (cont'd) (Unprocessed immunoblots (continued)**)**

Unprocessed immunoblots for indicated Figures panels.

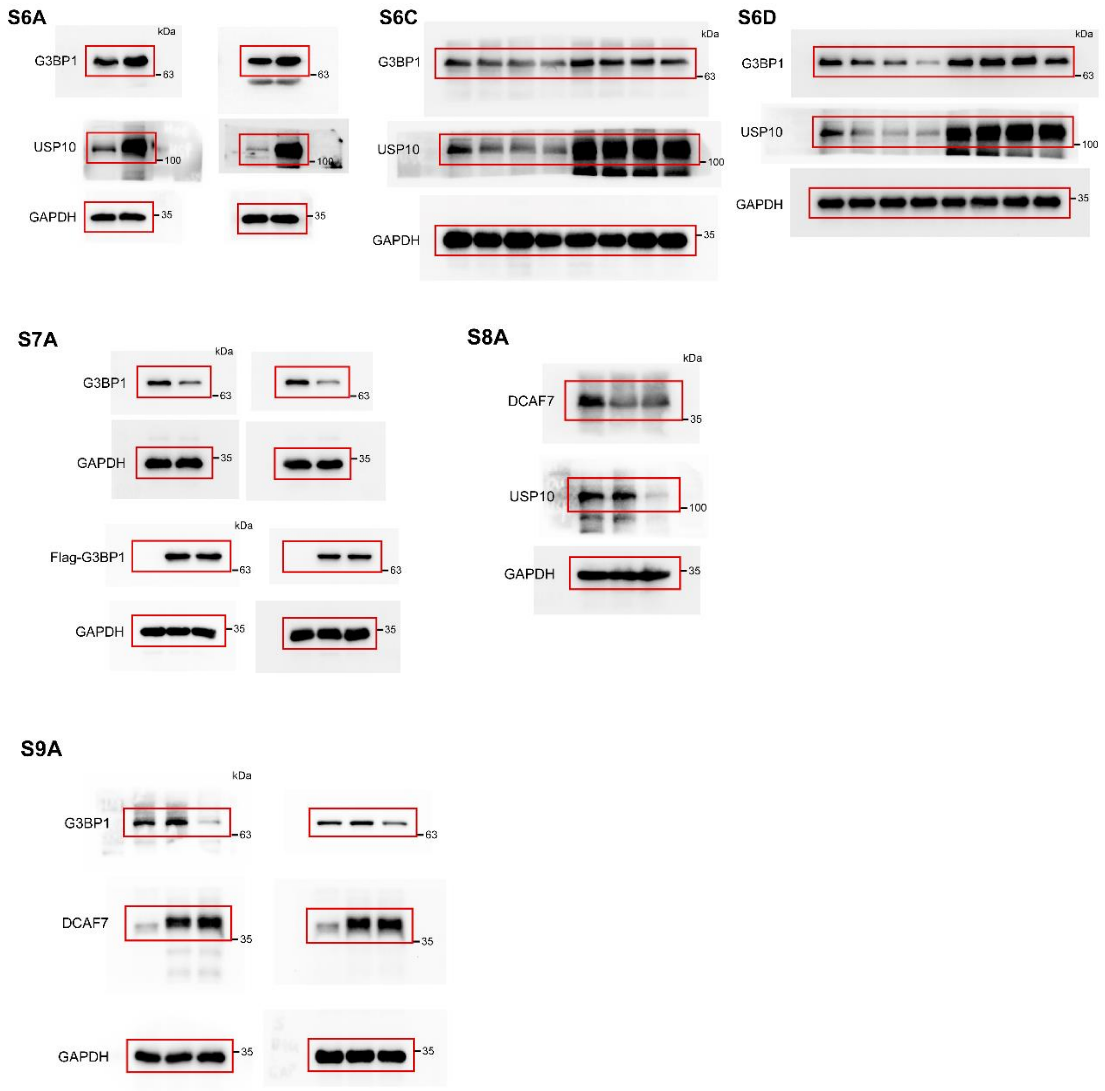

Figure S10. (cont'd) (Unprocessed immunoblots (continued)**)**

Unprocessed immunoblots for indicated Figures panels.

Table S1. Mass spectrometry analysis results for the anti-FLAG (FLAG-tagged DCAF7) immunoprecipitation complex.

Accession	-10lgP	Area	#Peptides	Description
sp|P23246|SFPQ_HUMAN	242.67	3.14E+08	27	Splicing factor proline- and glutamine-rich
sp|P08670|VIME_HUMAN	240.02	1.93E+08	26	Vimentin
sp|P11940|PABP1_HUMAN	217.58	8.81E+07	17	Polyadenylate-binding protein 1
sp|Q15233|NONO_HUMAN	210.28	1.06E+08	23	Non-POU domain-containing octamer-binding protein
sp|Q9UHB6|LIMA1_HUMAN	190.94	1.16E+08	17	LIM domain and actin-binding protein 1
sp|Q96PK6|RBM14_HUMAN	185.44	2.34E+07	11	RNA-binding protein 14
sp|P0CG39|POTEJ_HUMAN	180.89	7.97E+06	14	POTE ankyrin domain family member J
sp|Q13310|PABP4_HUMAN	174.29	9.21E+06	8	Polyadenylate-binding protein 4
sp|P61962|DCAF7_HUMAN	160.56	5.19E+07	8	DDB1- and CUL4-associated factor 7
sp|P20592|MX2_HUMAN	159.5	3.67E+07	12	Interferon-induced GTP-binding protein Mx2
sp|P51114|FXR1_HUMAN	158.71	3.67E+07	10	Fragile X mental retardation syndrome-related protein 1
sp|P52732|KIF11_HUMAN	153.21	2.26E+07	11	Kinesin-like protein KIF11
sp|P51116|FXR2_HUMAN	145.88	1.20E+07	8	Fragile X mental retardation syndrome-related protein 2
sp|Q13283|G3BP1_HUMAN	144.84	5.16E+07	9	Ras GTPase-activating protein-binding protein 1
sp|P52272|HNRPM_HUMAN	138.02	2.12E+07	8	Heterogeneous nuclear ribonucleoprotein M
sp|Q08211|DHX9_HUMAN	135.03	8.15E+06	5	ATP-dependent RNA helicase A
sp|Q96C19|EFHD2_HUMAN	130.73	1.16E+07	6	EF-hand domain-containing protein D2
sp|Q8WXF1|PSPC1_HUMAN	130.46	1.78E+07	7	Paraspeckle component 1
sp|P11021|BIP_HUMAN	129.46	1.23E+07	8	Endoplasmic reticulum chaperone BiP
sp|P14136|GFAP_HUMAN	122.89	8.74E+05	7	Glial fibrillary acidic protein
sp|Q15646|OASL_HUMAN	122.38	7.51E+06	7	2'-5'-oligoadenylate synthase-like protein
sp|P08174|DAF_HUMAN	121.18	6.24E+07	6	Complement decay-accelerating factor
sp|Q15025|TNIP1_HUMAN	111.39	3.47E+06	3	TNFAIP3-interacting protein 1
sp|P04899|GNAI2_HUMAN	109.5	6.88E+06	6	Guanine nucleotide-binding protein G(i) subunit alpha-2
sp|Q5JWF2|GNAS1_HUMAN	106.31	1.05E+07	5	Guanine nucleotide-binding protein G(s) subunit alpha isoforms Xlas
sp|Q14694|USP10_HUMAN	105.8	4.69E+06	4	Ubiquitin carboxyl-terminal hydrolase 10
sp|Q9NYL9|TMOD3_HUMAN	104.48	1.26E+07	3	Tropomodulin-3
sp|O14974|MYPT1_HUMAN	104.41	5.53E+06	5	Protein phosphatase 1 regulatory subunit 12A
sp|Q8IVT2|MISP_HUMAN	104.32	9.00E+06	5	Mitotic interactor and substrate of PLK1
sp|Q06787|FMR1_HUMAN	104.03	2.81E+06	5	Synaptic functional regulator FMR1
sp|O00160|MYO1F_HUMAN	101.65	3.74E+07	6	Unconventional myosin-If
sp|P60891|PRPS1_HUMAN	96.8	3.93E+06	2	Ribose-phosphate pyrophosphokinase 1
sp|P11908|PRPS2_HUMAN	96.8	3.93E+06	2	Ribose-phosphate pyrophosphokinase 2
sp|P21108|PRPS3_HUMAN	96.8	3.93E+06	2	Ribose-phosphate pyrophosphokinase 3
sp|P62805|H4_HUMAN	94.64	1.19E+07	3	Histone H4
sp|Q14573|ITPR3_HUMAN	93.5	4.22E+06	4	Inositol 1 4 5-trisphosphate receptor type 3
sp|O43854|EDIL3_HUMAN	91.67	7.30E+06	4	EGF-like repeat and discoidin I-like domain-containing protein 3
sp|P08754|GNAI3_HUMAN	90.27	4.36E+06	5	Guanine nucleotide-binding protein G(i) subunit alpha

sp|P07355|ANXA2_HUMAN	88.7	3.04E+06	2	Annexin A2
sp|Q92841|DDX17_HUMAN	87.9	1.42E+06	2	Probable ATP-dependent RNA helicase DDX17
sp|Q6YHK3|CD109_HUMAN	86.38	2.20E+06	5	CD109 antigen
sp|Q9Y3I0|RTCB_HUMAN	81.65	1.72E+06	2	RNA-splicing ligase RtcB homolog
sp|P21333|FLNA_HUMAN	77.37	1.56E+05	7	Filamin-A
sp|P29317|EPHA2_HUMAN	77.2	2.02E+06	2	Ephrin type-A receptor 2
sp|P38646|GRP75_HUMAN	76.41	1.86E+06	2	Stress-70 protein mitochondrial
sp|P62807|H2B1C_HUMAN	75.23	1.92E+07	3	Histone H2B type 1-C/E/F/G/I
sp|P0DP25|CALM3_HUMAN	73.85	2.04E+07	3	Calmodulin-3
sp|P0DP24|CALM2_HUMAN	73.85	2.04E+07	3	Calmodulin-2
sp|P0DP23|CALM1_HUMAN	73.85	2.04E+07	3	Calmodulin-1
sp|Q00839|HNRPU_HUMAN	73.79	1.92E+06	3	Heterogeneous nuclear ribonucleoprotein U
sp|P78386|KRT85_HUMAN	73.14	8.64E+07	5	Keratin type II cuticular Hb5
sp|P07910|HNRPC_HUMAN	72.43	4.76E+06	3	Heterogeneous nuclear ribonucleoproteins C1/C2
sp|Q13772|NCOA4_HUMAN	72.37	3.22E+06	3	Nuclear receptor coactivator 4
sp|Q15717|ELAV1_HUMAN	71.69	3.20E+06	2	ELAV-like protein 1
sp|Q9Y446|PKP3_HUMAN	70.33	3.24E+06	4	Plakophilin-3
sp|O75369|FLNB_HUMAN	68.77	4.48E+05	3	Filamin-B
sp|P84090|ERH_HUMAN	67.75	5.42E+06	2	Enhancer of rudimentary homolog
sp|P62280|RS11_HUMAN	65.32	3.46E+06	3	40S ribosomal protein S11
sp|Q3MHD2|LSM12_HUMAN	60.44	4.94E+05	1	Protein LSM12 homolog
sp|P60660|MYL6_HUMAN	59.89	6.93E+06	2	Myosin light polypeptide 6
sp|P19474|RO52_HUMAN	59.51	4.73E+06	3	E3 ubiquitin-protein ligase TRIM21
sp|O00571|DDX3X_HUMAN	57.55	2.32E+06	3	ATP-dependent RNA helicase DDX3X
sp|P67936|TPM4_HUMAN	57.53	1.70E+06	2	Tropomyosin alpha-4 chain
sp|O95425|SVIL_HUMAN	55.83	2.07E+06	3	Supervillin
sp|Q13751|LAMB3_HUMAN	55.35	3.97E+04	1	Laminin subunit beta-3
sp|P61978|HNRPK_HUMAN	55.23	2.39E+06	2	Heterogeneous nuclear ribonucleoprotein K
sp|P81605|DCD_HUMAN	53.23	1.69E+06	1	Dermcidin
sp|P13639|EF2_HUMAN	52.75	2.99E+06	2	Elongation factor 2
sp|Q14103|HNRPD_HUMAN	50.96	1.08E+06	2	Heterogeneous nuclear ribonucleoprotein D0
sp|P38159|RBMX_HUMAN	50.92	3.07E+06	2	RNA-binding motif protein X chromosome
sp|Q9BTC0|DIDO1_HUMAN	47.87	3.22E+06	2	Death-inducer obliterator 1
sp|P28799|GRN_HUMAN	46.91	3.48E+05	2	Progranulin
sp|Q92900|RENT1_HUMAN	46.87	1.33E+06	1	Regulator of nonsense transcripts 1
sp|Q5M775|CYTSB_HUMAN	45.1	6.05E+05	2	Cytospin-B
sp|P16104|H2AX_HUMAN	44.39	1.43E+06	1	Histone H2AX
sp|P0C0S5|H2AZ_HUMAN	44.39	1.43E+06	1	Histone H2A.Z
sp|Q71UI9|H2AV_HUMAN	44.39	1.43E+06	1	Histone H2A.V
sp|Q9BTM1|H2AJ_HUMAN	44.39	1.43E+06	1	Histone H2A.J
sp|P13010|XRCC5_HUMAN	43.42	7.53E+05	1	X-ray repair cross-complementing protein 5
sp|Q96HS1|PGAM5_HUMAN	43.33	2.96E+06	3	Serine/threonine-protein phosphatase PGAM5 mitochondrial
sp|P09874|PARP1_HUMAN	42.61	1.21E+06	1	Poly [ADP-ribose] polymerase 1
sp|Q14596|NBR1_HUMAN	42.38	1.55E+06	1	Next to BRCA1 gene 1 protein

sp|O15144|ARPC2_HUMAN	41.94	1.89E+06	1	Actin-related protein 2/3 complex subunit 2
sp|O15145|ARPC3_HUMAN	41.34	1.38E+06	1	Actin-related protein 2/3 complex subunit 3
sp|O95837|GNA14_HUMAN	40.01	4.09E+05	1	Guanine nucleotide-binding protein subunit alpha-14
sp|P29992|GNA11_HUMAN	40.01	4.09E+05	1	Guanine nucleotide-binding protein subunit alpha-11
sp|P50148|GNAQ_HUMAN	40.01	4.09E+05	1	Guanine nucleotide-binding protein G(q) subunit alpha
sp|Q92499|DDX1_HUMAN	39.94	6.46E+05	1	ATP-dependent RNA helicase DDX1
sp|P0DOX8|IGL1_HUMAN	39.29	1.99E+07	1	Immunoglobulin lambda-1 light chain
sp|B9A064|IGLL5_HUMAN	39.29	1.99E+07	1	Immunoglobulin lambda-like polypeptide 5
sp|Q7L2E3|DHX30_HUMAN	38.73	9.53E+05	1	ATP-dependent RNA helicase DHX30
sp|P60763|RAC3_HUMAN	38.67	1.23E+06	1	Ras-related C3 botulinum toxin substrate 3
sp|P15153|RAC2_HUMAN	38.67	1.23E+06	1	Ras-related C3 botulinum toxin substrate 2
sp|P63000|RAC1_HUMAN	38.67	1.23E+06	1	Ras-related C3 botulinum toxin substrate 1
sp|P13987|CD59_HUMAN	38.33	5.56E+06	1	CD59 glycoprotein
sp|P04792|HSPB1_HUMAN	36.31	1.39E+06	1	Heat shock protein beta-1
sp|P11279|LAMP1_HUMAN	36.3	5.33E+06	1	Lysosome-associated membrane glycoprotein 1
sp|Q13075|BIRC1_HUMAN	35.13	1.28E+08	2	Baculoviral IAP repeat-containing protein 1
sp|Q6WCQ1|MPRIP_HUMAN	34.09	2.14E+06	1	Myosin phosphatase Rho-interacting protein
sp|Q08999|RBL2_HUMAN	32.32	4.62E+06	2	Retinoblastoma-like protein 2
sp|Q71RC2|LARP4_HUMAN	30.39	6.12E+05	1	La-related protein 4
sp|P09382|LEG1_HUMAN	29.58	1.60E+06	1	Galectin-1
sp|P05161|ISG15_HUMAN	27.02	5.72E+05	1	Ubiquitin-like protein ISG15
sp|Q96L33|RHOV_HUMAN	26.49	1.14E+06	1	Rho-related GTP-binding protein RhoV
sp|Q06830|PRDX1_HUMAN	26.28	4.52E+05	1	Peroxiredoxin-1
sp|P32119|PRDX2_HUMAN	26.28	4.52E+05	1	Peroxiredoxin-2
sp|Q04837|SSBP_HUMAN	26.17	1.39E+06	1	Single-stranded DNA-binding protein mitochondrial
sp|Q96RK0|CIC_HUMAN	24.95	1.33E+06	1	Protein capicua homolog
sp|Q5HYA8|MKS3_HUMAN	23.92	3.92E+06	1	Meckelin
sp|Q9NZD8|SPG21_HUMAN	23.01	1.03E+06	1	Maspardin
sp|Q96DU9|PABP5_HUMAN	22.82	7.77E+02	1	Polyadenylate-binding protein 5
sp|Q2V2M9|FHOD3_HUMAN	22.71	6.23E+05	1	FH1/FH2 domain-containing protein 3
sp|Q15366|PCBP2_HUMAN	22.52	1.80E+06	1	Poly(rC)-binding protein 2
sp|Q10589|BST2_HUMAN	21.38	3.72E+06	1	Bone marrow stromal antigen 2
sp|Q9HCK8|CHD8_HUMAN	21.19	1.31E+06	1	Chromodomain-helicase-DNA-binding protein 8

Table S2. Correlations between DCAF7 expression levels and clinical features in Nasopharyngeal carcinoma patients.

Characteristic	Number of patients (%)		*P*-value*
	Low expression group (n = 117)	Low expression group (n = 78)	
Gender			
Male	91 (77.78%)	55 (70.51%)	0.252
Female	26 (22.22%)	23 (29.49%)	
Age (years old)			
< 60	101 (86.32%)	68 (87.18%)	0.863
≥ 60	16 (13.68%)	10 (12.82%)	
TNM stage†			
I/II	43 (36.75%)	28 (35.9%)	0.903
III/IV	74 (63.25%)	50 (64.1%)	
Distant metastasis			0.002
Yes	107 (91.45%)	59 (75.64%)	
No	10 (8.55%)	19 (24.36%)	
Disease			
Yes	92 (78.63%)	49 (62.82%)	0.016
No	25 (21.37%)	29 (37.18%)	
Death			
Yes	100 (85.47%)	56 (71.79%)	0.019
No	17 (14.53%)	22 (28.21%)	

* Pearson χ^2 test was used to compute the *P*-value.

† All patients were restaged based on the 7th edition of the AJCC Cancer Staging Manual.

Table S3 List of primers used in this study

RT-qPCR primers

Gene	Forward Primer (5′→3′)	Reverse Primer (5′→3′)
DCAF7	CACGGCAAACGGAAGGAGAT	GACGCCTTTTGTGTCAGGGA
G3BP1	AGCCTGTTCAGAAAGTCCTTAGC	CGAAGGCGATTATCTCGTCGGT
USP10	AAATGCCACCGAACCTATCGGC	CAGCCATTCAGACCGATCTGGA
Tubulin	CGTGTTCGGCCAGAGTGGTGC	GGGTGAGGGCATGACGCTGAA

Primers used for shRNA plasmid construction

Name	Primer (5′→3′)
sh-DCAF7#1-F	CCGGGTTCAGCTTGTTGGTTTAGATCTCGAGATCTAAACCAACAAGCTGAACTTTTTG
sh-DCAF7#1-R	AATTCAAAAAAGTTCAGCTTGTTGGTTTAGATCTCGAGATCTAAACCAACAAGCTGAAC
sh-DCAF7#2-F	CCGGGCTGGAGTGTTTGCTAAACAACTCGAGTTGTTTAGCAAACACTCCAGCTTTTTG
sh-DCAF7#2-R	AATTCAAAAAGCTGGAGTGTTTGCTAAACAACTCGAGTTGTTTAGCAAACACTCCAGC
sh-G3BP1#1-F	CCGGCGGGAATTTGTGAGACAGTATCTCGAGATACTGTCTCACAAATTCCCGTTTTTG
sh-G3BP1#1-R	AATTCAAAAACGGGAATTTGTGAGACAGTATCTCGAGATACTGTCTCACAAATTCCCG
sh-G3BP1#2-F	CCGGCCACCTCATGTTGTTAAAGTACTCGAGTACTTTAACAACATGAGGTGGTTTTTG
sh-G3BP1#2-R	AATTCAAAAACCACCTCATGTTGTTAAAGTACTCGAGTACTTTAACAACATGAGGTGG
sh-USP10#1-F	CCGGCGACAAGCTCTTGGAGATAAACTCGAGTTTATCTCCAAGAGCTTGTCGTTTTTG
sh-USP10#1-R	AATTCAAAAACGACAAGCTCTTGGAGATAAACTCGAGTTTATCTCCAAGAGCTTGTCG
sh-USP10#2-F	CCGGCCTATGTGGAAACTAAGTATTCTCGAGAATACTTAGTTTCCACATAGGTTTTTG
sh-USP10#2-R	AATTCAAAAACCTATGTGGAAACTAAGTATTCTCGAGAATACTTAGTTTCCACATAGG

sgRNA sequences

Name	Sequence (5′→3′)
sg-DCAF7	GCGGCGACAAACAACGACGA
sg-USP10	TCCATCGACTGCCAGTACCC

siRNA sequence

Name	Sequence (5′→3′)
si-G3BP1	CCCGTAAGAAGGAATGTTA

Primers used for overexpression plasmid construction

Name	Primer (5′→3′)
Flag-DCAF7-F	GGAATTCGCCACCATGTCCCTGCACGGCAAACGGA
Flag-DCAF7-R1	ATCCTTGTAATCCACTCTGAGTATCTCCAGGC
Flag-DCAF7-R2	GACTAGTCTACTTATCGTCGTCATCCTTGTAATCCACTC TGA
Flag-G3BP1-F	GGAATTCGCCACCATGGTGATGGAGAAGCCTAGTCCCC TG
Flag-G3BP1-R1	ATCCTTGTAATCCTGCCGTGGCGCA
Flag-G3BP1-R2	GACTAGTTCACTTATCGTCGTCATCCTTGTAATCCTG
Flag-G3BP1(K36R)-F	CTGCATAGATTTTATGGAAGGAACTCTTCTTATGTCCAT GGGGGA
Flag-G3BP1(K36R)-R	TCCCCCATGGACATAAGAAGAGTTCCTTCCATAAAATC TATGCAG
Flag-G3BP1(K76R)-F	AACTTCACCAACTGCCACACCAGGATTCGCCATGTTGA TGCTCAT
Flag-G3BP1(K76R)-R	ATGAGCATCAACATGGCGAATCCTGGTGTGGCAGTTGG TGAAGTT
Flag-G3BP1(K123R)-F	GCTCCTGAGGGGTCTGTTGCAAATAGATTCTATGTTCA CAATGAT
Flag-G3BP1(K123R)-R	ATCATTGTGAACATAGAATCTATTTGCAACAGACCCCTC AGGAGC
Myc-USP10-F1	GGAATTCCATATGGCCACCATGGCCCTCCACAGCCCGC AGTATA
Myc-USP10-F2	TTTATGGGTGACATGCCCCCGTCAGTTACGCCCAGGAC TTGTAAC
Myc-USP10-R1	GTTACAAGTCCTGGGCGTAACTGACGGGGGCATGTCA CCCATAAA
Myc-USP10-R2	GACTAGTTTACAGATCCTCTTCAGAGATGAGTTTCTGC TCCAGCAGGTCCACTCGGCG
Myc-USP10(C424A)-F1	GGAATTCCATATGGCCACCATGGCCCTCCACAGCCCGC AGTATA
Myc-USP10(C424A)-F2	GGAACTGGGCCTACATTAATGC
Myc-USP10(C424A)-R1	GCATTAATGTAGGCCCAGTTCC
Myc-USP10(C424A)-R2	GACTAGTTTACAGATCCTCTTCAGAGATGAGTTTCTGC TCCAGCAGGTCCACTCGGCG
